# The environmental impacts of one of the largest tailing dam failures worldwide

**DOI:** 10.1038/s41598-017-11143-x

**Published:** 2017-09-06

**Authors:** Vanessa Hatje, Rodrigo M. A. Pedreira, Carlos Eduardo de Rezende, Carlos Augusto França Schettini, Gabriel Cotrim de Souza, Danieli Canaver Marin, Peter Christian Hackspacher

**Affiliations:** 1 Centro Interdisciplinar de Energia e Ambiente (CIENAM), Instituto de Química, Universidade Federal da Bahia. Rua Barão de Jeremoabo, s/n, Ondina, 40170-115 Salvador, BA Brazil; 2 Laboratório de Ciências Ambientais, Centro de Biociências e Biotecnologia, Universidade Estadual do Norte Fluminense. Av. Alberto Lamego 2000, 28015-620 Campos dos Goytacazes, RJ Brazil; 3 Laboratório de Hidrodinâmica Costeira, Departamento de Oceanografia, Centro de Tecnologia e Geociências, Universidade Federal de Pernambuco. Av. Prof. Moraes Rego, 1235, 50910-000 Recife, PE Brazil; 4 Observatório FG do Semiárido Nordestino, Faculdade Guanambi. Avenida Pedro Felipe Duarte, 4911, São Sebastião, 46430-000 Guanambi, BA Brazil; 5 Laboratório de Geoquímica Isotópica, Departamento de Petrologia e Metalogenia, Instituto de Geociências e Ciências Exatas, Universidade Estadual Paulista, Av. 24A,1515, 13506-900 Rio Claro, SP Brazil

## Abstract

The impacts of the SAMARCO iron tailing spill along more than 650 km, between the dam and the plume of the Doce River in the Atlantic, were assessed by the determination of toxic metals. The tailing spill caused a substantial increase in suspended sediment loads (up to 33,000 mg L^−1^), in addition to large depositions of waste along the Doce basin. The highest estimated transport of dissolved metals was observed for Fe (58.8 μg s^−1^), Ba (37.9 μg s^−1^) and Al (25.0 μg s^−1^). Sediments reached the highest enrichment factors (EFs) for Hg (4,234), Co (133), Fe (43), and Ni (16), whereas As (55), Ba (64), Cr (16), Cu (17), Mn (41), Pb (38) and Zn (82) highest EFs were observed for suspended particulate matter (SPM). Iron, As, Hg, Mn exceeded sediment quality guidelines. Therefore, the risk of occurrence of adverse effects is highly possible, not only due to the dam failure, but also due to the Fe mining and the artisan Au mining. Heavy rain episodes will likely cause enhanced erosion, remobilization, and transport of contaminated particles, sustaining high inputs of SPM and metals for the years to come and threatening the ecosystem services.

## Introduction

Most of the elements currently used in industrial applications, technology development, and energy generation are produced through the mining, extraction, and processing of mineral ores. Mining activities produce several footprints due to changes in the landscape, with the mobilization of large amounts of rocks and soils, high demand of water and energy and effluents associated with tailing production and disposal. Historically, mining can be considered one of the main anthropogenic activities that contribute to major and trace elements pollution of river basins worldwide, which may lead to serious human health implications and also long-term impairment to waterways and biodiversity. There are two serious problems usually associated with mining activities, the acid-mine drainage^1^ and the mine tailings that contribute deliberately and/or accidentally to the burden of anthropogenically derived metals to river basins.

What differs tailing dam accidents and other anthropogenic sources of metals is the amount and velocity which slurry may travels, covering soils, river sediments, floodplains, riverbanks, and damaging water quality and killing biota. According to Lewin and Macklin^2^ tailing wastes may have a passive dispersion, *i.e*. tailing is transported alongside the natural sediment load of a river in a manner that does not disrupt natural systems, or they may undergo an active transformation. In the latter, the whole fluvial system is transformed through the input of wastes causing a dramatic increase in sediment supply and significant changes in the morphology of river basins. The long-term effects of dam failures have been found to depend on four factors^3^: (1) the quantity and (2) characteristics of the waste, (3) the rate at which waste is discharged into a river system and (4) the effectiveness of cleanup procedures. According to Macklin and colleagues^4^, the failures of Aznalcóllar and Porco dams represent end-members of a spectrum of environmental impacts in river basins due to mining activities. The breach of the dam of a pyrite mine in Aznalcóllar, Spain, caused the release of 4 × 10^6^ m^3^of toxic tailings^5^. The fast construction of a retention dam minimized the damage of the toxic wastes, enriched in Zn, Pb, Cu, As, Sb, Cd, and Tl, to rivers and the National Park of Donãna^6^. Although cleanup operations were efficient to remove around 90% of the tailing material deposited in the river channel^7^, as showed by an evaluation performed 10 years after the tailing spill^8^, it created a highly unstable river channel and floodplain susceptible to accelerated erosion^9^. El Porco accident, on the other hand, although had contaminated around 300 km of a river system, did not alter the erosion and sedimentation processes of the Pilaya River basin^10^.

**Figure 1:**
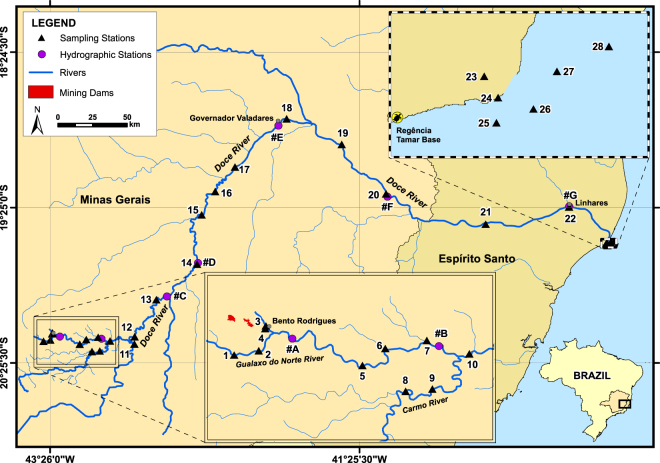
Sample locations along Doce Riven Basin, Minas Gerais, and Espírito Santo states, Brazil. This figure was prepared using ArcGIS (ArcMap 10.1; http://desktop.arcgis.com/).

The November 2015 spill at the Samarco Mineração SA, was one of the largest ever reported failure of a tailing dam^11^ that illustrate the increasing rate of serious tailing failures. It has been estimated that more than 35 million m^3^ of mining residues slid down a mountainside when Fundão tailing dam (SAMARCO) failed, causing the death of 19 people, and transforming more than 650 km of rivers (Gualaxo do Norte, Carmo and Doce rivers; Fig. 1), which were the primary source of water and food for several communities, in a “sea” of red mud. At least 1,500 ha of natural reserves and Krenak indigenous land were adversely impacted^12^. The tailing slurry also reached the mouth of the Doce River, in the Atlantic Ocean, where a pronounced reddish plume was observed.

The aim of this study was to assess the fluxes and spatial distribution of major and trace elements along the 650 km between the area of the Samarco dam failure and the plume of Doce River in the Atlantic Ocean. Water and sediments and suspended particulate material (SPM) were characterized, bioavailability and enrichment factors were estimated to fill the knowledge gap of the detrimental impacts caused by the dam burst. Discrimination was made between the impact caused by SAMARCO dam accident and the historic mining contamination. Finally, overall environmental implications were also outlined.

## Results

### Ancillary variables

Waters were well oxygenated in most stations, the pH measurements varied between 7.10 and 8.25. In general, pH values were slightly higher than historic values (6.9–7.2)^13^. Temperature varied from 23.0 °C (#5) to 29.7 °C (#19). Saline waters were observed only at stations #25 (16.3), #26 (1.25), #27 (10.8), #28 (32.2).

**Figure 2:**
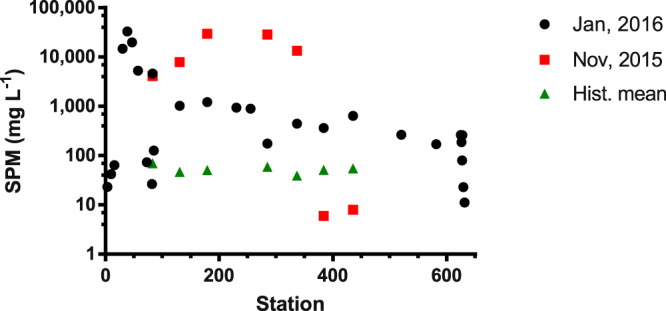
Distribution of suspended particulate material (SPM) in Doce River basin. Squares and triangles represent, respectively, data collect 5 days after the accident and historic means (IGAM, 2015).

Concentrations of SPM reached values up to 33,000 mg L^−1^ at Gualaxo do Norte (#6) and decreased exponentially until the plume reached the Atlantic Ocean (11.2 mg L^−1^; Fig. 2). Sediment samples were poorly-sorted and could be classified as sandy silt or silty sand (Supplementary Fig. S1).

### Trace and major elements

#### Water

Carmo River control stations (#8 and #9; Supplementary Table S1) presented the highest concentrations of dissolved As (1.79 µg L^−1^), Cr (2.49 µg L^−1^), Pb (10.1 µg L^−1^), and relatively high concentrations of Ni (1.74 µg L^−1^), Al (23.0 µg L^−1^), and Fe (68.7 µg L^−1^), despite being considered a control area for this study, once they were not in the route of the tailing slurry main flow. Similarly, Gualaxo do Norte control sites (#1 and #2) presented the highest concentration of Ni (2.55 µg L^−1^), and relatively high contents of Pb (0.77 µg L^−1^), Cr (1.27 µg L^−1^), Mn (124 µg L^−1^), and Cu (0.51 µg L^−1^). Cobalt highest concentration (0.88 µg L^−1^) was observed in a creek station close to the dam accident. Comparatively to other stations, Piranga (#11) presented high concentrations of Th (0.019 µg L^−1^), Fe (80.7 µg L^−1^), and Cu (1.56 µg L^−1^). Among the control stations only Mn, at the station #3, presented concentration above recommended screening values (80 µg L^−1^)^14^.

**Figure 3:**
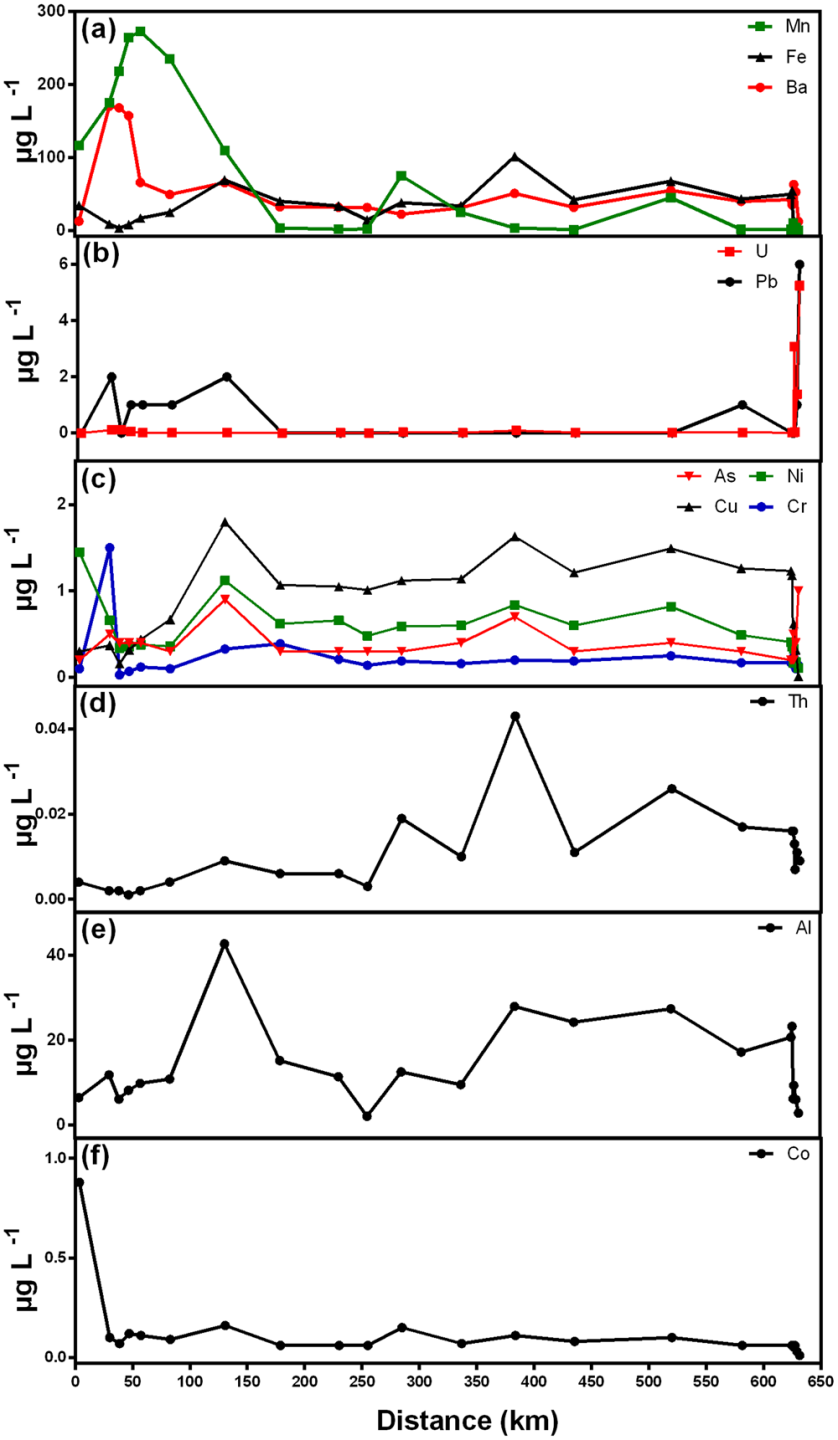
Concentrations of dissolved trace elements along the Doce River downstream Samarco dam. Only the stations along the flow of the tailing slurry are presented.

Dissolved element concentrations did not show a clear dilution pattern from the closest stations to the tailing dam failure seaward (Fig. 3). Several elements presented concentration peaks along the Gualaxo do Norte River and Doce rivers (#13, #19, #21, and #28). Among the determined elements, Ba presented concentrations above recommended acute benchmark for surface freshwater (110 µg L^−1^)^14^ at the most upstream stations of Gualaxo do Norte. Concentrations of Al (Stations # 13, 19, 20, 21, 23 and 24) and Mn (Stations # 5–7, 10, 12 and 13) in Doce River were above recommended Brazilian standards (20 µg L^−1^, 100 µg L^−1^, respectively)^15^.

All dissolved elements, with the exception of Mn, presented an increase in transport, and flux peaks along the Doce River basin (Supplementary Fig. S2). The highest estimated transport, at the Doce River mouth, was observed for Fe (58.8 μg s^−1^), Ba (37.9 μg s^−1^) and Al (25.0 μg s^−1^).

#### Suspended Particulate Material

The highest concentrations of all elements, except Zn, were observed in Carmo River (Supplementary Table S2). Arsenic presented concentration below detection limits (0.035 µg g^−1^) for almost all sites, with the exception of control sites #8 (286 µg g^−1^), #9 (78.0 µg g^−1^) that presented concentrations above Upper Effects Threshold (UET, 17 µg g^−1^), value above which toxicity is predicted^14^. Iron and Mn also presented concentrations above UET (4% and 1,100 µg g^−1^, respectively)^14^ at station #8, whereas Cd, Cr, Cu, Ni, Pb, and Zn presented concentrations above Lower Threshold Level (TEL)^14^. At the Piranga River control station (#11), the level of Fe was higher than UET, and concentrations of Cd and Cu were above TEL values (0.597 µg g^−1^ and 35.7 µg g^−1^ respectively)^14^.

**Figure 4:**
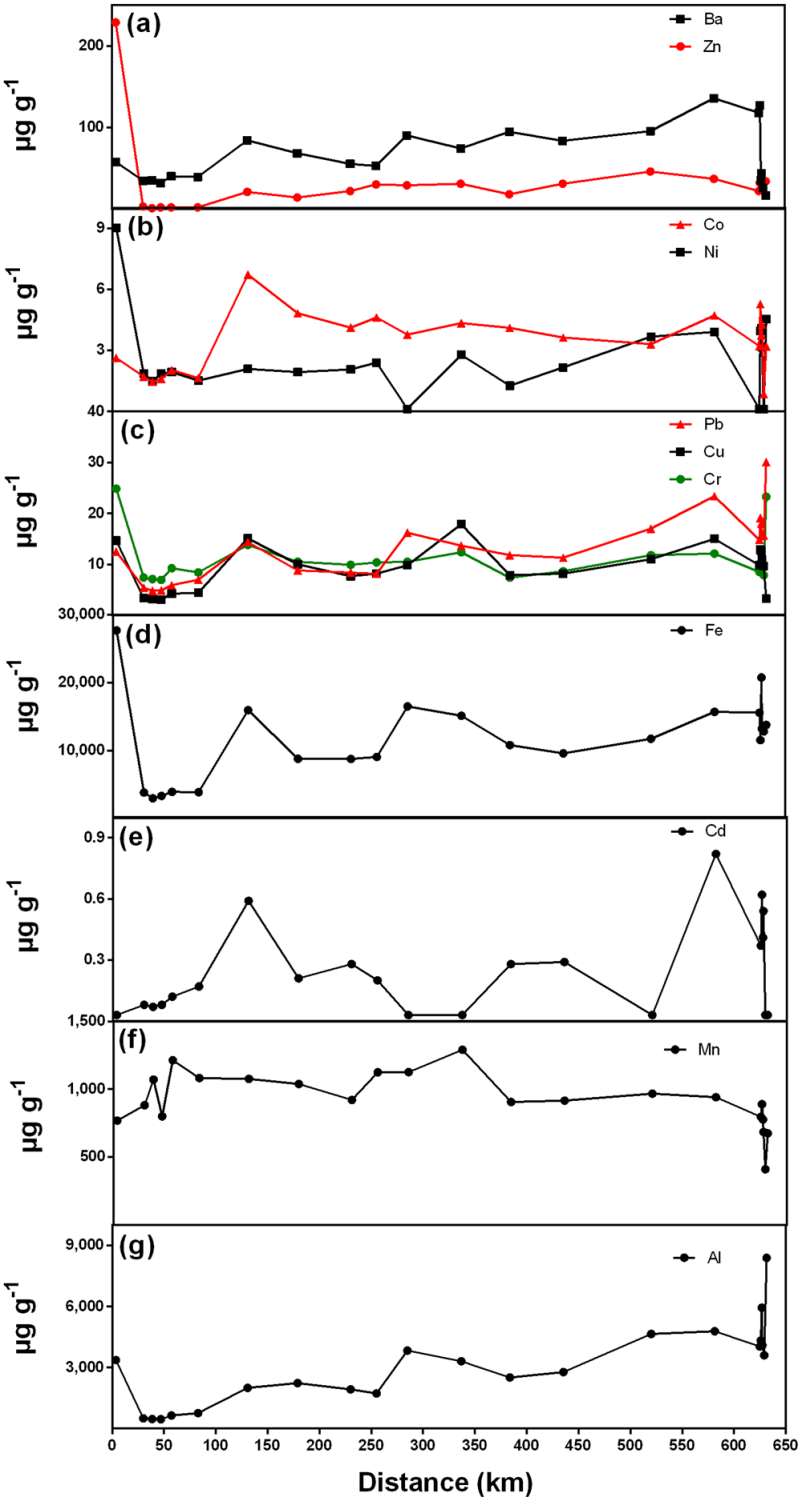
Concentrations of particulate trace elements along the Doce River downstream Samarco dam. Only the stations along the flow of the tailing slurry are presented.

Along the path of the tailing slurry (Fig. 4), a maximum was observed in the concentrations of particulate Cr, Fe, Ni and Zn in the closest station to the dam failure (#3). Concentrations of most elements stayed low until a peak was observed at station #13 (131 km downstream the dam) for Ba, Cd, Co, Cr, Cu, Fe, and Pb, similarly to what was observed for the dissolved phase. From the upper Doce River seawards, most particulate elements concentrations presented small spatial variability, with only small peaks. In general, particulate trace metals fluxes (Supplementary Fig. S3) displayed a slight increase along Doce River basin until station # 20, then the transport of all particulate elements decreased seawards. Along the Doce River, the displayed minimum fluxes of particulate metals coincided with the low level of suspended particulate material.

#### Sediments

Pseudo-total fraction in bulk sediments (Supplementary Table S3) presented high levels of As, Cd, Cr, Fe, Ni and Mn in all control stations. Arsenic, Cd, and Fe levels were above UET values for all control stations (17 µg g^−1^, 3 µg g^−1^, and 4%, respectively)^14^; Mn levels were above UET (1100 µg g^−1^)^14^ for stations #1, 2, 8 and 9; Ni concentrations were above UET values (75 µg g^−1^)^14^ for stations #1 and 2; Cr concentrations were above TEL for stations #2, 8, 9, and 11 and above UET for stations #1 (17.3 and 95 µg g^−1^, respectively)^14^. Mercury presented the highest values at stations #1 and 2.

**Figure 5:**
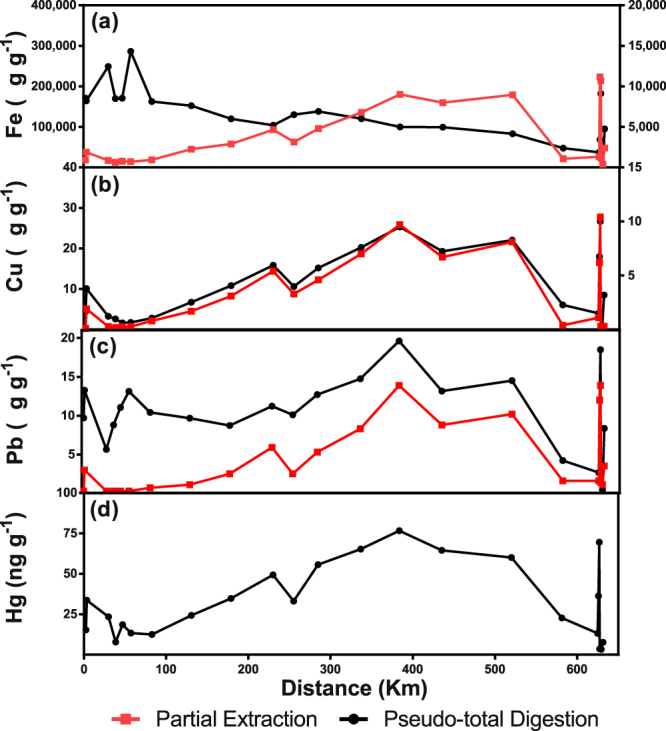
Bioavailable and pseudo-total concentrations of selected metals along the Doce River downstream Samarco dam. Only the stations along the flow of the tailing slurry are presented. For Fe and Cr, pseudo-total digestion concentrations are shown on the left y-axis and bioavailable fraction in the right y-axis.

Concentrations of pseudo-total metals in bulk sediments along the Doce River system displayed 3 distribution patterns (see examples in the Fig. 5). Cadmium and Fe presented the highest concentrations in the most upstream stations, which peaks in stations #5 and #10 followed by a decrease seaward, with another peak at the mouth of the Doce River. For most elements, a “bell-shaped” distribution, *i.e*., higher concentrations in the middle of the river than in most upstream and downstream stations, was observed. Cobalt presented the third pattern, with the lowest concentrations in the middle of the Doce River. Concentrations of Cd and Cr, for most stations, were above UET (Cd = 3 µg g^−1^)^14^ and TEL (Cr = 37.3 µg g^−1^)^14^, respectively. For several stations, the concentrations of Ni and some values of Mn were also above sediment quality criteria (Ni-TEL = 18 µg g^−1^, Mn-UET = 1100 µg g^−1^)^14^.

In general, the concentration of elements for the bioavailable fraction followed the same pattern that the concentrations obtained by the pseudo-total fraction (Fig. 5). Cadmium and Fe were exceptions; the bioavailable fraction along the Doce River showed an increase in concentrations seaward. In average, the metals that presented the highest bioavailable fraction in decreasing order were Co > Mn > Pb > Ba > Cu > Zn > Ni > Cr > Al > Cd > Fe. The highest bioavailable fraction for several elements occurred in control stations.

### Principal Component Analysis

**Figure 6:**
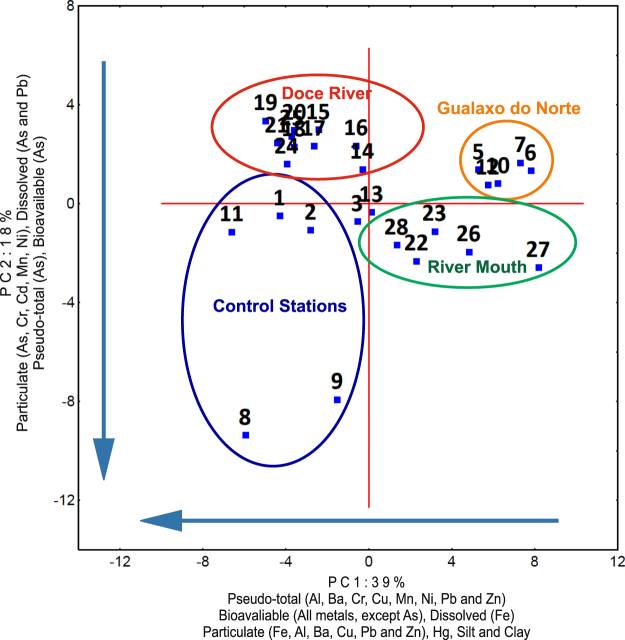
Principal component analysis of water, suspended particulate material and sediment samples along Doce River basin.

Two principal components (PC; Fig. 6 and Supplementary Table S4) explained 57% of the total dataset variability. Silt, clay, Hg, all elements in the bioavailable fraction of sediments (except As and Co), most elements in the pseudo-total fraction (Al, Ba, Cr, Cu, Mn, Ni, Pb, and Zn), dissolved Fe, and particulate Fe, Al, Ba, Cu, Pb, and Zn presented high negative loadings for PC1 (39% of the total variance). The PC2 (17.7% of the total variability) was mainly composed by As in dissolved, particulate and sedimentary phases, besides Cd, Cr, Mn, and Ni in the particulate phase and Pb in the dissolved phase.

## Discussion

### SPM fluxes after the accident

Despite all transport, dilution and sedimentation processes affecting slurry distribution and concentrations, the dam burst was still an important source of suspended particulate material for the system almost 3 months after the accident. SPM concentrations were still at least two orders of magnitude higher than the historic average (Fig. 2)^13^. The slurry input promoted an active transformation of the fluvial system^2^, causing readily observable erosion and degradation along its flow and intense floodplain aggradation in several areas.

The estimated suspended sediment flux (SSF) along the east Brazilian (from Rio Grande do Norte State southwards), Uruguayan and Argentinean coast is of about 120.10^9^ ton year^−1^ ^16^, and the Doce River contribution was estimated as of 11.10^6^ ton year ^−1^ ^17^. The SSF of the Doce River is relatively high when compared with other Brazilian basins with similar size (*e.g*., Paraíba do Sul River, area of 55,500 km^2^ and SSF of 4.10^6^ ton year^−1^ ^17^; Guaíba River, area of 85,000 km^2^ and SSF of 1.10^6^ ton year^−1^ ^18^). The higher SSF value is directly related to the soil use in the basin, and it is very likely that mining activities in the basin have an important contribution to that. The specific case of the dam breakage produced an enormous SPM anomaly in the upper Doce River, strongly attenuated by the dilution downstream. However, large amounts of sediments of the initial slurry flood wave were trapped in the riverbanks and dam reservoirs along the river. This material is now promptly available to be eroded and transported after strong rain storms followed by competent discharges in years to come. It is expected that in the SPM concentration in the middle and lower basin would return to its pre-dam breakage levels under low river discharge periods. On the other hand, very high SPM concentration will occur associated with flood events. The trapped sediment will be transported through short term pulses^2, 19, 20^ when the hydrodynamics in the delta area will favor the direct delivery into the adjacent ocean^21^.

Doce River inner shelf has been characterized as a supply regime dominated by the input of suspended fine sediments through the river mouth and the formation of a terrigenous muddy deltaic lobe south of the river mouth^22, 23^. Flocculation processes due to increase in salinity seem to be playing an important role in the plume region and accumulation of fine sediments in the shelf. However, because this is a wave-dominated environment^24^, it promotes the offshore transport of fine sediments that mostly accumulates in areas deeper than 10 m depth^23^. The dam failure has changed this pattern and promoted an increase in the deposition of fine sediments close to shore, in shallower areas. It is expected that during winter, the arrival of cold fronts, with higher energy waves, could remobilize and transport northwards the poorly consolidated fine material originated from the tailing dam. The input of fine sediments to the watershed and the coastal zone promoted a massive mortality of biota due to burial and suffocation by mud. Of special concern are the damaging effects in extremely rare jelly fish species such as the gender Kishinouyeacorbini that has its distribution overlapped by the dispersion pattern of the tailing plume^25^. The burial of benthic organisms, followed by changes in taxonomical and functional diversity has been previously described as short-term and long-term impacts of mine tailing inputs to the environment^26, 27^.

### Mining activities and impacts in Doce River basin

The Quadrilátero Ferrífero is a well-known mineral deposit, being one of the most important mining regions of Brazil. Iron, Al, and Au have been mined in the region at least since the 17^th^ century. Whereas artisan small-scale mining dominated the mining activities for a long period of time, modern large-scale operations started in the second half of the 20^th^ century and were intensified since the beginning of the 1980s by multinational enterprises with large infrastructure and facilities, such as SAMARCO. Mineral assemblages of Au and Fe ores are rich in potentially toxic elements, such as As, Mn, and Hg^28, 29^. Positive anomalies of trace elements in the regions are expected to result from both the lithology, naturally enriched in metals, and mining and smelting activities, which promotes the mobilization of metals and increases the fluxes of these elements to soils, atmosphere, and water column.

### Control stations: the impacts of artisan small-scale mining

To evaluate the impact of the SAMARCO dam failure in the concentration of trace elements, several sites were selected as controls, *i.e*., areas that were not in the main pathway of the tailing spill and, as such, the trace element contents in these stations are not expected to be related to the slurry. The control stations were clearly separated from other sites in the PCA (Fig. 6) mostly due to their levels of As concentrations in sediments, dissolved and particulate phases. Arsenic provenance is likely related to small-scale Au mining activities. It is ubiquitous the presence of As in gold ores in the Quadrilátero Ferrífero. In the Carmo River basin, Au deposits are found in the contact rocks from Nova Lima group and Minas Supergroup^30, 31^. These deposits contain major amounts of arsenopyrite, either in quartz and carbonate veins or disseminated in iron formation^32^. Among the Au deposits, the As/Au ratio varies from 300 to 3,000 in the Au-bearing sulfites^33^. Oxidation of these sulfites can then release As to water bodies, soils and sediments where they can be immobilized by adsorption into Fe, Mn, and Al oxi-hydroxides or in clay minerals^34^. It has been estimated that more than 390,000 tons of As enriched tailing have been disposed in the Quadrilátero Ferrífero drainage system^30^. Weathering of mine tailings and smelting activities are a major source of As for the region.

Previous studies have found high levels of As in sediments (≈20–2,000 mg kg^−1^) and water bodies in Quadrilátero Ferrífero related to both natural and anthropogenic sources^28, 30, 35–38^. Potential health risks to human populations were also suggested due to the high levels of As (2.2–106 µg L^−1^) found in urine samples of children^39^. Concentrations above UET levels for particulate As in Carmo River and for sediments in Carmo, Gualaxo do Norte, and Piranga rivers are predictive of adverse impacts to biota and deserve attention. Arsenic presents in water and sediments may be incorporated in fish that become an important pathway for the As exposition to humans.

One of the collateral damages of Au artisan mining well documented in the literature is the environmental contamination due to Hg amalgamation^40, 41^. Corroborating to the hypothesis that Au mining is causing enrichment of As, Carmo River also displayed the highest Hg concentrations (85–480 ng g^−1^). It has been previously showed that Hg in Carmo River occurs mostly in the elemental form, possibly due to its association to gold mining activities^42^.

Particle size is an important controlling factor for the concentration, mobility, migration, and transformation of trace elements in sediments^43^. Fine sediments have high specific surface areas, negative charge and also high clay and organic matter contents. The high percentage of fine sediments in station #1 (91%), compared to #2 (24%), in Carmo River, indicated that granulometry is playing an important role in the adsorption and cationic exchangeable capacity of sediments. In station #2, it is expected that Hg presents higher mobility, possibly as Hg^2+^ produced from the oxidation of Hg^0^, and transported downstream associated to SPM and also with organic ligands. The comparison of Hg levels to toxicity thresholds (TEL, 0.174 µg kg^−1^)^14^ indicates that sediments of Carmo River present concentrations probably at toxic levels.

Lead contamination has also been related to Au mining. In Quadrilátero Ferrífero, both As and Pb occur associated with sulfide-rich veins and may be anthropogenic enriched by mining activities^36^. Moreover, in Fe ore deposits Pb can also be present associated to galena. Concentrations of Pb in control stations, however, did not indicate human driven impacts.

Carmo, Gualaxo do Norte, and Piranga control stations did not show signals of impacts of the dam burst in neither SPM nor Fe concentrations. However, Hg and As, two important tracers of the adverse effects of artisan gold mining, were clearly enriched in sediments. These results indicate that clandestine, artisan small-scale gold mining is still present in the Carmo and Gualaxo do Norte basin and contribute to the contamination of the environmental compartments to levels that may have adverse effects on ecosystems services. Clearly, Au mining operators and environmental authorities poorly implement environmental regulations and sustainable practices.

### Effects of the dam failure on the distributions of trace and major elements

The solid residue from the iron ore processing at the headwaters of the Doce River basin presents 57% of Fe, 14% of SiO_2_, 7.7% of H_2_O_2_ and 1.3% of Al^44^. More recently, a study showed that the slurry from the SAMARCO dam has a particle size from 1 to 200 µm, and it is largely composed of SiO_2_, Fe, Mn, Ca and Cr^45^. Preliminary results indicated that Fe in the tailing is predominantly in its trivalent state and that the mineralogy of Fe (oxy)hydroxides is characteristic of the presence of hematite (Hatje *et al*. unpublished).

The high levels of the Fe in the pseudo-total fraction of sediments (Fig. 5) showed that the dam failure promoted Fe enrichment to levels that potentially impact the quality of the benthic environment. Concentrations of Fe observed in sediments in Gualaxo do Norte were up to 3.5-fold the background level (8.2%)^35^. A decrease of up to 6-fold was observed in Fe pseudo-total contents from the closest station to the dam seaward, where it increased again. Whereas the percentage of bioavailable Fe remained relatively constant and low for the Gualaxo do Norte River (≈0.4%), an increase was observed along the Doce River reaching up to 10% of the pseudo-total fraction at the lower Doce River and 16% at the estuary mouth. This gradual increase indicates that Fe is been mobilized and becoming more bioavailable along its transport along the river.

Maximum concentrations of Fe associated to SPM (4.8%) under dry conditions, almost 3 months after the tailing accident, were below background concentrations (8.2%)^35^. This may indicate that the elevated Fe contents, which are highly reactive, directly linked to the tailing solid residue, were rapidly removed from the water column and are mostly stored in floodplain and sediments. During heavy rain episodes, erosion may readily remobilize Fe and other metals from sediments causing enhanced transport further downstream of contaminated particles from the tailings and streambed.

Fe and most of other elements in dissolved, particulate and in sediments, together with the fine fraction of sediments and SPM were associated in the PCA (Fig. 6). The PC1 showed a gradient of contaminants from areas with relatively lower metal concentrations and high sand contents (river/estuary mouth) to the sites most impacted by metals (control stations). The association of this myriad of elements and sample positions along the PC1 suggests that not only the spill of the dam failure, but several anthropogenic activities combined are impacting on the downstream river. Corroborating to that, relatively high concentrations of several metals were also observed scattered along Doce River, despite the relative strong dilution and increasing distance from the dam tailing.

It is important to emphasize that there are more than 2 hundred municipalities in the Doce watershed that together discharge untreated sewage of a population of more than 2 million^46^. Nutrient discharge and damming of the river has caused successive blooms of cyanobacteria in river waters^47^. Economic growth, centered on commodity exploitation, puts pressure on the natural resources of the Doce River basin, where there are important mineral reserves, besides agroindustry, and intense urban and industrial development. Source apportionment for the elements in such cases is difficult and very complex. Concentration peaks of several elements were observed in the middle Doce River near Governador Valadares, Ipatinga, and Linhares. Although granulometry plays an important role controlling metal distributions, point sources seem to be the determinant factor here. The decreasing profile of Fe and Cd concentrations in sediments indicated that mining slurry had an impact in these elements distributions. But for most of the other elements, other anthropogenic sources such as industries, sewage, mining practices and agriculture are also contributing to the distribution patterns observed.

To compensate the differences in mineralogy and grain size variability along sediment samples, trace elements were normalized by Al^48^. Background trace elements levels of sediments (Table 1) from the Doce river basin had been previously determined (Fe^35^ all other elements^49^) and were used to estimate the enrichment factors (EF). The EFs were estimated as the ratio between metal concentration and background level (Al normalized). The most contaminated sites, located in the Gualaxo do Norte in the closest stations to the SAMARCO dam, reached EFs of 43 for Fe, 35 for Ba, 5.8 for Cu, 9.1 for Cr, 16 for Ni, and 37 for Zn (Table 1

**Table 1:** Enrichment Factors (EF) for trace metals in sediments of the Doce river basin.

**Station**	**Fe**_**PT**_	**Fe**_**SPM**_	**As**_**PT**_	**As**_**SPM**_	**Ba**_**PT**_	**Ba**_**SPM**_	**Co**_**PT**_	**Co**_**SPM**_	**Cr**_**PT**_	**Cr**_**SPM**_	**Cu**_**PT**_	**Cu**_**SPM**_	**Mn**_**PT**_	**Mn**_**SPM**_	**Ni**_**PT**_	**Ni**_**SPM**_	**Pb**_**PT**_	**Pb**_**SPM**_	**Zn**_**PT**_	**Zn**_**SPM**_	**Hg**
1	1.8	0.6	2.5	—	2.4	**64**	2.1	1.0	4.2	7.8	1.2	8.8	2.1	**26**	8.4	7.6	2.4	**11**	1.6	**28**	**286**
2	3.2	0.9	7.7	—	**11**	**49**	**13**	0.8	4.0	**16**	5.7	**11**	6.5	**41**	9.6	2.1	4.0	**29**	3.8	**36**	**4234**
3	4.1	1.8	—	—	1.9	6.9	**23**	0.3	3.3	4.8	2.6	9.8	0.5	3.2	7.1	6.0	5.2	**13**	4.2	**82**	**363**
4	**14**	#	—	#	3.8	#	**28**	#	5.4	#	3.9	#	1.4	#	**11**	#	**13**	#	**12**	#	**558**
5	**43**	1.7	—	—	**35**	**28**	**93**	1.4	8.2	9.7	5.8	**15**	8.4	**25**	**16**	8.5	**15**	**38**	**37**	6.3	**1735**
6	**29**	1.4	—	—	4.7	**30**	**133**	1.3	8.8	9.9	4.6	**15**	1.8	**32**	7.6	7.3	**24**	**36**	**21**	3.4	**577**
7	**18**	1.6	—	—	3.0	**28**	**77**	1.4	2.4	9.8	1.7	**15**	1.2	**24**	2.6	9.1	**18**	**37**	**12**	4.0	**828**
8	1.0	1.0	9.3	**55**	1.9	8.0	8.4	—	2.5	—	1.8	8.0	0.9	0.3	3.3	—	1.7	—	2.2	0.0	**116**
9	2.9	0.8	**23**	**32**	2.9	9.0	**36**	—	4.6	—	2.6	9.0	1.9	0.3	4.7	—	3.0	—	4.1	0.0	**143**
10	**43**	1.3	—	—	4.3	**25**	**126**	1.3	9.1	9.4	2.7	**15**	1.6	**27**	**12**	6.9	**31**	**32**	**28**	3.6	**860**
11	0.1	0.9	1.1	—	0.8	7.9	2.0	0.8	0.8	2.1	1.0	8.9	0.1	2.6	1.3	1.5	0.5	**11**	1.3	7.5	**41**
12	**11**	1.1	—	—	2.1	**21**	**60**	0.9	2.5	7.2	1.9	**13**	0.9	**20**	2.5	4.5	**11**	**32**	9.0	3.5	**351**
13	3.3	1.8	—	—	1.7	**17**	8.6	1.4	2.1	4.5	1.5	**17**	0.6	7.5	2.5	2.4	3.3	**25**	4.1	**13**	**230**
14	1.4	0.9	—	—	1.5	**12**	5.9	0.9	1.4	3.0	1.3	**10**	0.4	6.5	2.1	2.0	1.7	**14**	2.4	7.3	**180**
15	0.7	1.0	—	—	1.2	**12**	3.3	0.9	1.1	3.3	1.1	9.0	0.3	6.7	1.6	2.4	1.2	**15**	1.8	**14**	**142**
16	1.4	1.1	—	—	1.3	**12**	3.2	1.1	1.4	3.9	1.2	**11**	0.3	9.1	1.8	3.1	1.7	**16**	2.5	**21**	**154**
17	0.9	0.9	—	—	1.0	9.4	2.3	0.4	0.9	1.8	1.0	5.8	0.3	4.1	1.3	—	1.3	**15**	1.8	9.1	**151**
18	0.5	1.0	—	—	1.0	9.0	1.9	0.5	0.7	2.4	0.9	**12**	0.2	5.4	1.1	1.9	1.0	**14**	1.4	**11**	**118**
19	0.3	0.9	—	—	1.0	**15**	1.5	0.7	0.6	1.9	0.7	7.1	0.2	5.0	0.8	1.1	0.9	**16**	1.2	8.5	**94**
20	0.4	0.8	—	—	1.1	**12**	1.9	0.5	0.7	2.0	0.9	6.7	0.3	4.6	1.0	1.8	0.9	**14**	1.4	**13**	**125**
21	0.3	0.6	—	—	1.1	8.2	1.6	0.3	0.6	1.6	0.8	5.3	0.2	2.9	0.9	1.8	0.8	**13**	1.3	**12**	**89**
22	1.6	0.7	—	—	1.7	**11**	**26**	0.4	2.2	1.6	2.1	7.1	0.8	2.7	2.1	1.8	2.3	**17**	4.6	9.4	**338**
23	0.9	0.8	—	—	1.1	**12**	**21**	0.3	1.5	1.4	1.0	5.5	0.4	2.8	1.4	—	1.0	**13**	2.7	6.7	**133**
24	0.2	0.6	—	—	0.4	**12**	1.2	0.5	0.5	1.5	0.4	6.7	0.2	2.9	0.7	2.1	0.4	**15**	0.8	9.0	**37**
25	0.4	0.8	—	—	0.5	2.4	1.1	0.3	0.5	1.1	0.6	4.8	0.1	1.8	0.8	1.5	0.7	**11**	1.0	6.9	**70**
26	0.8	0.7	—	—	1.2	4.2	**25**	0.4	1.3	1.4	1.1	6.0	0.3	2.3	1.5	1.7	1.3	**13**	2.2	6.7	**49**
27	0.4	0.8	—	—	1.0	2.9	**65**	0.1	1.2	1.4	0.6	6.0	0.4	1.6	1.2	—	—	**15**	2.3	9.0	**97**
28	1.4	0.4	—	—	0.9	0.8	**11**	0.2	1.9	1.8	1.3	0.9	0.4	1.1	1.8	1.2	1.9	**12**	3.4	4.9	**48**
Background^+^	8.2		8.8		45		2.1		28		8.0		1.290		8.0		5.2		15		0.19

EFs higher than 10 are presented in bold.

Where: — = not calculated, see Tables S2 and S3; # = SPM sample was not collected for this station; ^+^Fe %^35^; other elements µg g^−1^ ^49^.

). In most cases, the metal EFs decreased by up to one order of magnitude downstream. This decline cannot be explained by changes in granulometry, which presented a general increase in the fine fraction towards the river mouth. With the exception of Fe, Ni, and Co, most metals along the Doce river basin presented EFs for SPM higher than for sediments. Besides, peaks in EFs for SPM were also observed for Pb, Cr, Ni, Mn and Ba at the headwaters of Gualaxo do Norte. These anomalous high EFs indicate that mining operations, besides the dam failure, are contributing to the total loading of suspended particulate materials enriched in Ba, Cr, Cu, Mn, Pb and Zn (EFs: 64, 16, 11, 41, 8, 29, 36, respectively).

Mercury displayed high EF (37–4,234) in all studied sites along the Doce River watershed, reflecting more than 300 years of gold exploitation. The highest EF was observed in the headwaters of Gualaxo do Norte (EF: 4,234) and Carmo River (EF: 1,735). Similarly to other trace elements, there was a decreasing trend for Hg EFs along the Doce River system. Based on the toxicity characteristics of Hg, transfer, and bioaccumulation along the food change, it is very likely the incidence of adverse biological effects in the study area due to Hg alone.

### Metals partitioning

Iron partition coefficients (K_d_) were in the order of 10^5^ and tended to slightly decrease from Gualaxo do Norte towards Doce River mouth. During transport, trace element sorption and dilution may result in a reduction of the dissolved phase and these affect the overall transport of all elements. In coastal waters, the increase in pH (7.2–7.9) and salinity (0–32) affected the stability of Fe oxides and promote colloidal aggregation and coagulation increasing K_d_ from 10^5^ to 10^7^.

**Figure 7:**
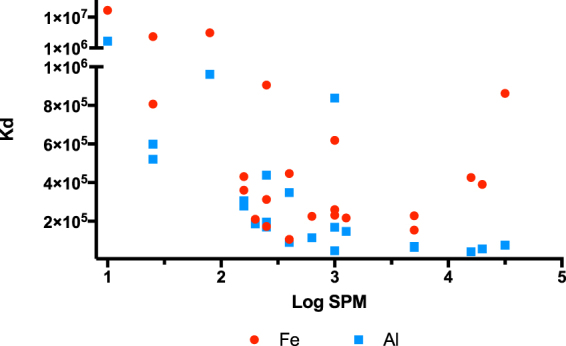
Partitioning Coefficient (K_d_) as a function of suspended particulate concentrations (log SPM concentrations) for the Doce River watershed.

Suspended particulate material is a master variable controlling scavenging of trace elements, and hence the K_d_ of most elements. It is expected that an enhancement in SPM concentrations, promotes an increase in the removal of metals if surface process controls the overall removal from the aqueous phase rather than a precipitation mechanism^50^. Contrary to this expectation, K_d_ for all elements, except Cr, displayed a decline with increasing SPM concentrations, as exemplified in Fig. 7, despite the very high contents of SPM observed in the Doce River watershed. This anomalous behavior, named particle concentration effect (PCE), has been observed in marine and freshwater systems^51–53^. The PCE may be a consequence of the commonly use of 0.45 µm pore filtration to separate dissolved and particulate phases that disregards the colloidal phase, producing a procedure artifact. It is expected that the higher the concentration of SPM, the higher would be the concentration of colloids. Future studies in Doce River should discriminate dissolved from particulate fractions considering the colloidal phase to reduce PCE. The major (Fe, Al, and Mn) and several trace elements possibly exist to a significant extent in the colloidal phase, but this hypothesis has yet to be tested.

### Environmental implications

Brazil has had several environmental challenges, many of them related to disaster management systems. Government agencies have tried to manage all kinds of environmental accidents without a solid coordination with stakeholders^54^. Consequently, the environmental impacts worsened once Brazil failed to follow best practices of emergency and disaster management. The SAMARCO dam failure is just one example where a clear gap between preparedness and response lead to the death of people, the destruction of houses, government buildings, and farmlands, the mortality of biota, changes in macrobenthic assemblages, interruption of energy and water supplies, and fisheries ban. The tailing slurry changed drastically the Doce River watershed and caused several environmental services come to a halt.

The cause of the dam collapse it is still unknown. The failure of the SAMARCO tailing dam was preceded by a small-magnitude seismic sequence within the mine area, around the time of the collapse^55^. However, there are no cases in the literature that connect dam collapses and small magnitude earthquakes^55^. More than a year after the accident, the definitive cause of the collapse has yet to be resolved. Several aspects have been evocated including foundation failure or bad impoundment management, fast increase of tailing deposition, poor management, and human inadequate practices.

Regardless the causes of the dam collapse, there is a gigantic amount of Fe ore waste to be dealt. Although the composition of the tailing slurry is mainly Fe and Si oxides^44, 56^, toxicity tests revealed that mud and soil present potential cytotoxicity and DNA damage^45^. This study did not include toxicity tests. However, the levels of Fe, As, Hg, Mn and other elements observed here, when compared to screening tools for environmental assessments (*e.g*., TEL and UET), exceeded recommended values in several stations. Therefore, the risk of occurrence of adverse effects is highly possible; not only due to the dam failure, but also the small scale Au mining in Carmo River basin, and Fe mining in the headwaters of Gualaxo do Norte. The prospect of contaminated soils in flood plains and streambed sediments being resuspended and transported downstream and possibly onshore can not be neglected on the view of the amount of tailing material deposited on the riverbanks along hundreds of kilometers in the Doce River watershed. That may happen when solids are remobilized during the high-energy rainy period, producing high turbidity waters, associated with metal contaminants that may persist for long periods due to the high amount of source material accumulated in the Doce River basin. Moreover, the trawling-induced continuous stirring of the upper layers of sediments in the fishing grounds in the shelf nearby the Doce River mouth may lead to a continuous addition of sediments, increasing the turbidity and acting as a source of several major and trace elements for the coastal waters.

The long lasting effects of this accident are still unknown and an appropriate in depth study for the years to come needs to be accomplished. The questions will keep coming, and the research community and funders must continue to pursue them, with speed, strength and the highest urgency to mitigate environmental damages and restore the functioning of ecosystems.

## Material and Methods

### Study Area

The SAMARCO, located near the Gualaxo do Norte (Fig. 1), has been mining Fe ores since the 1970’s. The beneficiation of the Fe ore includes: comminution, classification, desliming, concentration and flotation^57^. After all these processes, the slurry is transported by a pipeline (400 km) to the pellet plant, where the pulp and pellets are produced. The production capacity is estimated in 30 million ton y^−1^ ^58^. The dam breakage (on November 5^th^, 2015, at −20.231384 S and −43.443065 W) took place in the Gualaxo do Norte River, a tributary of the Carmo River, which by its turn debouches into the Doce River. The Doce River drainage basin has nearly 84,000 km^2^ located in the Minas Gerais (MG, 86%) and Espírito Santo (ES, 14%) States. The Doce River (Fig. 1) runs around 650 km before reaching the Atlantic Ocean. There are 4 hydroelectric power dams along the river. The Doce basin is subject to a tropical savannah and wet tropical climate with temperatures above 18 °C. The rainy period is concentrated between October to March, with the maximum in January. The averaged precipitation rate is of about 1,200 mm year^−1^ ^59^. The mean Doce River discharge is of 900 m^3^ s^−1^, ranging from 250–300 m^3^ s^−1^ during the dry period to 1,800 m^3^ s^−1^ at January, on averaged terms. Flood peaks usually exceed 5,000 m^3^ s^−1^, and maximum flood records can reach 9,000 m^3^ s^−1^. The flood peaks last for one or two days. The Doce River watershed supports a highly diverse economy, which includes agricultural, industrial (steel and paper mill, metallurgy, chemical, etc.) and mining (Fe, Mn, and Au). Despite all anthropogenic developments in the Doce River basin, important and diverse natural ecosystems are still found in the region.

### Sampling

Sediments, SPM and water were collected from Gualaxo do Norte, Carmo, Piranga and Doce rivers in summer 2016 (75 days after the accident). Twenty-eight sampling sites were selected in order to cover the whole river extension (~650 km) downstream from the dam burst to the Doce River plume, in the Atlantic Ocean (Fig. 1). Tailing mud (#4; Fig. 1) was also collected 3 km from the accident, in the Bento Rodrigues village and in a stream nearby (#3). The samples collected in Carmo (# 8 e 9), Piranga (#11) and Gualaxo do Norte (#1 and #2) were not located in the main direction of the slurry flow and were considered control sites for the accident. Water temperature, pH, salinity, turbidity and dissolved oxygen measurements were conducted *in situ* using a calibrated multi-parameter water quality meter (Horiba U-50, Horiba, Japan).

Water samples were collected in 1 L, pre-acid cleaned (10% HNO_3_), plastic bottles and filtered through pre-weighted 0.45 μm white cellulose acetate membranes. Water samples were acidified with pH 2 (HCl 30% Suprapure®, MERCK, Germany) and stored in double plastic bags and kept at 4 °C until analysis. Sediment samples were collected manually from the river bed and divided into two parts, the first used for the determination of particle-size distribution and physical characterization, and the second for chemical analyses. Sediments were kept frozen until analysis.

### Sample preparation and analysis

Sediments were freeze-dried, homogenized and comminuted in a ball mill (8000D, SPEX Sample Prep, USA). For bulk sediments (250 mg) a ‘pseudo-total’ digestion was performed using aqua regia (3 mL concentrated HCl + 1 mL concentrated HNO_3_) in Teflon Parr bombs, for 16 h at a temperature of 120 ± 5 °C. Additionally, an extraction using 1 M HCl (20 mL), shaking for 12 h at room temperature, was carried out with sediments and SPM samples^60^. This dilute acid leach solubilized only the more readily bioavailable fractions and left behind residual metals within the structure of silicate minerals. The latter extraction is reported to closely correlate with biological availability^61^, and hereafter is called as the bioavailable fraction.

Trace and major elements in sediments and suspended particulate material were determined by ICP OES (Model 720 ES, Varian, Australia), whereas dissolved metals in water samples were determined by HR-ICP-MS (ELEMENT 2, Thermo Scientific, Germany). The characteristics and settings of the HR-ICP-MS and the ICP OES are presented in Supplementary Tables S5 and S6, respectively.

The HR-ICP-MS was optimized using a tune solution containing Li, In and U (1 ng ml^−1^). The isotopes^137^Ba,^208^Pb,^232^Th,^235^U were measured in low resolution mode,^27^Al,^52^Cr,^55^Mn,^56^Fe,^59^Co,^60^Ni,^63^Cu were analyzed in medium resolution and^75^As was determined in high resolution. A standard solution containing 3 ng ml^−1^ of Sc, Ge, In and Bi was used as internal standards for the HR-ICP-MS measurements.

The Hg (253.652 nm) was determined from a sub-aliquot of 0.5 g dry sediment by adding 8 mL of aqua regia. The extracts were taken to a Microwave (Mars Xpress, CEM, USA), for 25 minutes at a temperature of 95 °C and power of 1,600w^62^. After cooling, the final extract was filtered on Whatman 40 filter paper and made to 50 ml with ultrapure water in a volumetric flask. The determination of Total Hg was performed by the Hg Analyzer (Quick Trace M-7500, Teledyne Cetac Technologies, USA). The analytical performance revealed a recovery of 91% for Hg.

The precision and accuracy of the analytical techniques were assessed using a marine sediment CRM (MESS-3, National Research Council of Canada, Canada), and a river water CRM (SLRS-6, National Research Council of Canada, Canada). Blanks and replicates were analyzed with each batch of samples. Recoveries for MESS-3 varied between 60 to 91%, for Cr and As, respectively, for pseudo-total digestions and 3.6 to 66%, for Al and Pb, respectively, for the HCl extractions. River water SLRS-6 recoveries ranged from 95% (Fe) to 116% (Mn) (Supplementary Table S1).

For grain size analysis, sediments were first sieved through a 2 mm mesh and 0.5 mm, to separate the gravel and coarse sand fractions. The fraction <0.5 mm was then analyzed by a Particle Size Analyzer with Laser Diffraction (model 1064, Cilas Particle Size, USA). SysGran (version 3.0, M. Camargo, Brazil; 200.17.232.45/sysgran) was used to classify samples.

### Dilution model and transport

A model was elaborated (Supplementary Table S7) to investigate the dilution process from the dam burst area all the way to the river mouth. The river discharge data, obtained from the Brazilian Water Agency (ANA, http://hidroweb.ana.gov.br/), from the only two available stations in the Gualaxo do Norte River: #A - Bicas and #B -Fazenda Ocidente, the former at 11.6 km downstream from the dam, and five stations along the Doce River: #C - Fazenda Cachoeira D’Antas, #D - Cachoeira dos Óculos, #E - Governador Valadares, #F - Resplendor and #G - Linhares, the latter the closest gauge station from the river mouth (~40 km; Fig. 1), were used in the dilution model. To minimize small-scale variability, monthly averages were used with the aim of constructing a mean hydrological scenario for the sampling period. The stations #A and #G (Fig. 1) are strategically positioned, once they reflect the flow regime at the dam area and the proximity of the river mouth, respectively. However, the former was decommissioned in 1966, and the latter in 1996. The longer lasting station in operation is the #B - Fazenda Ocidente, commissioned in 1938 and still operating (Supplementary Table S8).

The dilution factors *D* along the river course was empirically modeled (Supplementary Fig. S4), by a best fit to a polynomial function of distance *l* as:

1 $$D(1)={al}^{3}+{bl}^{2}+cl+d$$

where the coefficients are: a = −1.17e-06; b = 1.34e-03; c = 2.99e-02; d = 0.35, and the coefficient of determination r^2^ = 0.96 (Supplementary Fig. S4A). The dilution factor for each sampling station (Supplementary Table S7) was obtained by Eq. (1).

In order to assess the possibility of introducing bias in the dilution model due to the different periods of operation of each gauge station, correlation coefficients were estimated between each station and #B (Supplementary Table S8). In all cases the correlations were significant (p < 0.05), indicating that the hydrological regime of the basin responded uniformly at monthly basis. The synoptic mean discharges ratio to station #B (Qs; Supplementary Table S8) were slightly different from the overall mean discharge for all stations (Q), although the major difference was only of 0.5% for #E. Despite the fact that the time series are not synoptic, the proportion of the mean discharge is about the same when normalized to the #B, which was composed by a synoptic record of at least 20 years. The ratio between the mean discharge at each station to the #A -Bicas, most upstream station, provided the dilution factors (Q_S_/Q_S-A_; Supplementary Table S8). The volume contribution of the #A-Bicas station was estimated to be around 1/250 of the total discharge of the entire Doce River drainage basin.

To verify and validated the dilution model, Eq (1) was applied to calculate the dilution of the SPM concentrations and the result was compared with the best fit of the SPM along the river (Supplementary Fig. S4B). The best model to describe the SPM along the river was an exponential decay function of distance from the dam as:

2 $$SPM{(l)}_{fit}=a\,ex{p}^{bl}+c\,ex{p}^{dl}$$

where the coefficients are: a = 5.039; b = −1.39 10^−02^; c = 7.06; d = -4.18 10^−04^, and the coefficient of determination r^2^ = 0.92. The modeled SPM was compared with the estimated SPM, and the best adjustment was achieved with a SPM of 57,000 mg L^−1^ at *l* = 0. The comparison was made by the mean ratio of the modeled (Eq. 1) to the estimated (eq. 2; Supplementary Fig. S4) SPM values. The dilution model derived represented satisfactorily the spatial distribution of the SPM, hence, increasing the confidence of the model to calculate the transport of properties.

The transport of the elements associated with the SPM, T_SPM_, was calculated by the product of the element concentration (kg kg^−1^), the SPM concentration (kg m^−3^), and the mean river discharge (m^3^ s^−1^). The transport of the elements dissolved in water, T_w_, were calculated by the product between the element concentration (kg kg^−1^), mean river discharge (m^3^ s^−1^) and the water density (kg m^−3^), taken as standard 1,000 kg m^−3^. For the transport, it was used the estimated SPM concentration instead of the observed one. The reasoning for that was to minimize the natural SPM noisy.

### Statistical analysis

Principal component analyses (PCA) were performed using concentrations of trace elements in the dissolved and particulate phases, and in sediments, and ancillary data (*e.g*., granulometry and SPM concentrations). To account for the different units of the variables, the data was log (x + 1) transformed (except ancillary variables), normalized and Euclidean distance were used for Principal Component Analysis (PCA). Analyses were performed using STATISTICA, version 10, StatSoft Inc, USA.

### Data availability

All data generated or analyzed during this study are included in this published article (and its Supplementary Information file).

## Electronic supplementary material

supplementary material
