# Supplementary material for: The environmental impacts of one of the largest tailing dam failures worldwide
Source: Sci Rep. 2017 Sep 6;7:10706. doi: 10.1038/s41598-017-11143-x (PMC5587546; doi:10.1038/s41598-017-11143-x)
Supplement: Supplementary file 1 — supplementary material [file 41598_2017_11143_MOESM1_ESM.doc]

**Supplementary Material**

**The environmental impacts of one of the largest tailing dam failures worldwide**

Vanessa Hatje^1^*, Rodrigo M. A. Pedreira^1^, Carlos Eduardo de Rezende^2^, Carlos Augusto França Schettini^3^, Gabriel C. de Souza^4^, Danieli Canaver Marin^5^, Peter Christian Hackspacher^5^

^1^Centro Interdisciplinar de Energia e Ambiente (CIENAM), Instituto de Química, Universidade Federal da Bahia. Rua Barão de Jeremoabo, s/n, Ondina, 40170-115, Salvador, BA, Brazil

^2^Laboratório de Ciências Ambientais. Centro de Biociências e Biotecnologia. Universidade Estadual do Norte Fluminense. Av. Alberto Lamego 2000, 28015-620, Campos dos Goytacazes, RJ, Brazil

^3^Laboratório de Hidrodinâmica Costeira. Departamento de Oceanografia, Centro de Tecnologia e Geociências, Universidade Federal de Pernambuco. Av. Prof. Moraes Rego, 1235, 50910-000, Recife, PE, Brazil

^4^Observatório FG do Semiárido Nordestino, Faculdade Guanambi, Avenida Pedro Felipe Duarte, 4911 - São Sebastião, 46430-000, Guanambi, BA, Brazil

^5^Laboratório de Geoquímica Isotópica. Departamento de Petrologia e Metalogenia, Instituto de Geociências e Ciências Exatas. Universidade Estadual Paulista. Av. 24 A,1515, 13506-900 - Rio Claro, SP, Brazil


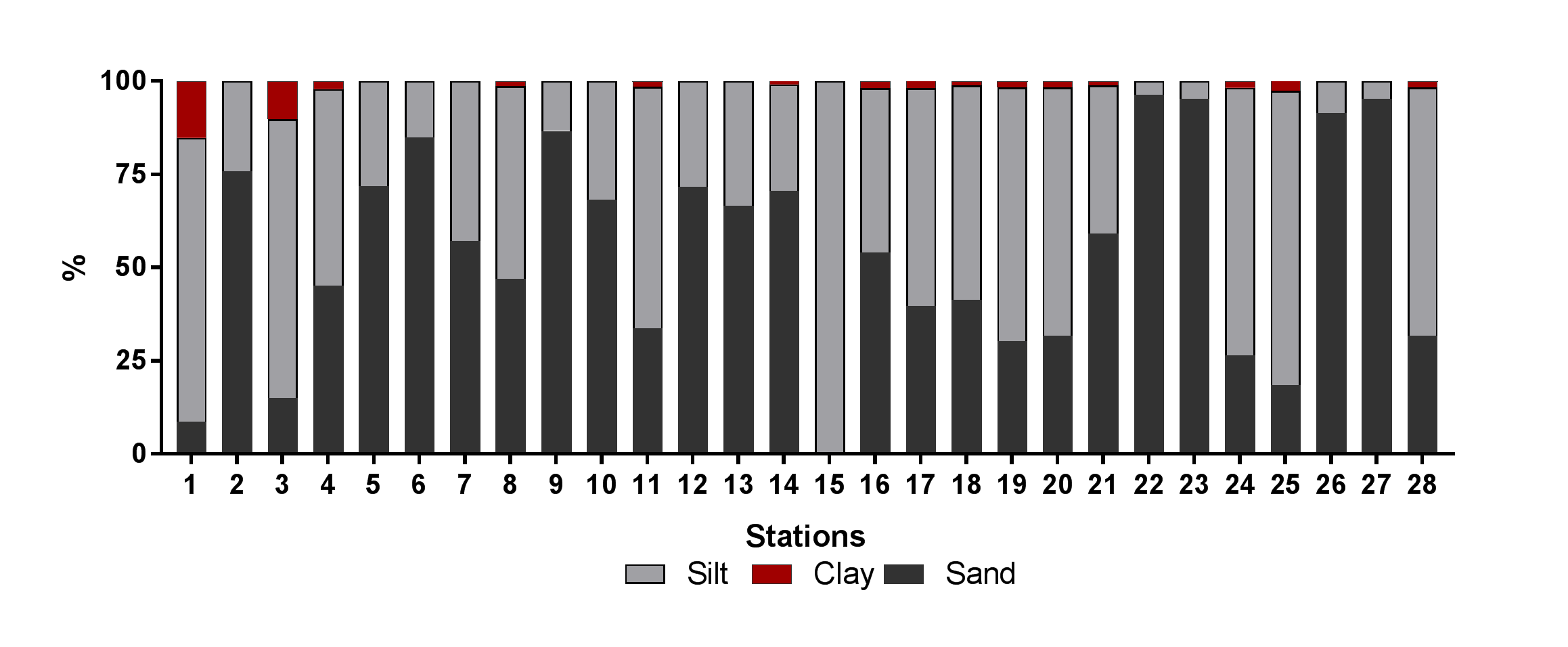


**Figure S1.** Grain size distribution for sediment samples along Doce river basin.


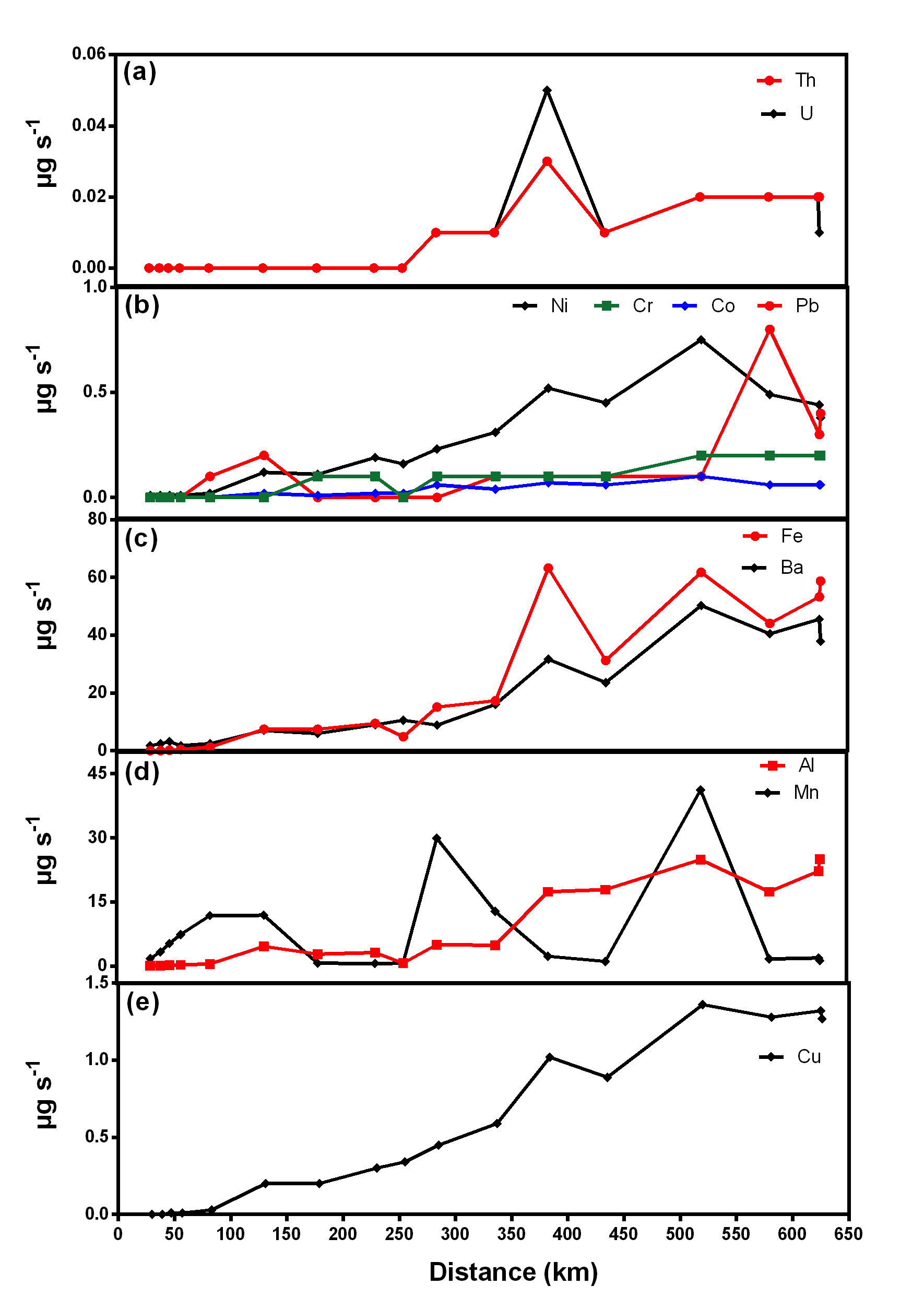


**Figure S2.** Fluxes of dissolved trace elements along the Doce river downstream Samarco dam. Only the stations along the flow of the tailing slurry are presented.


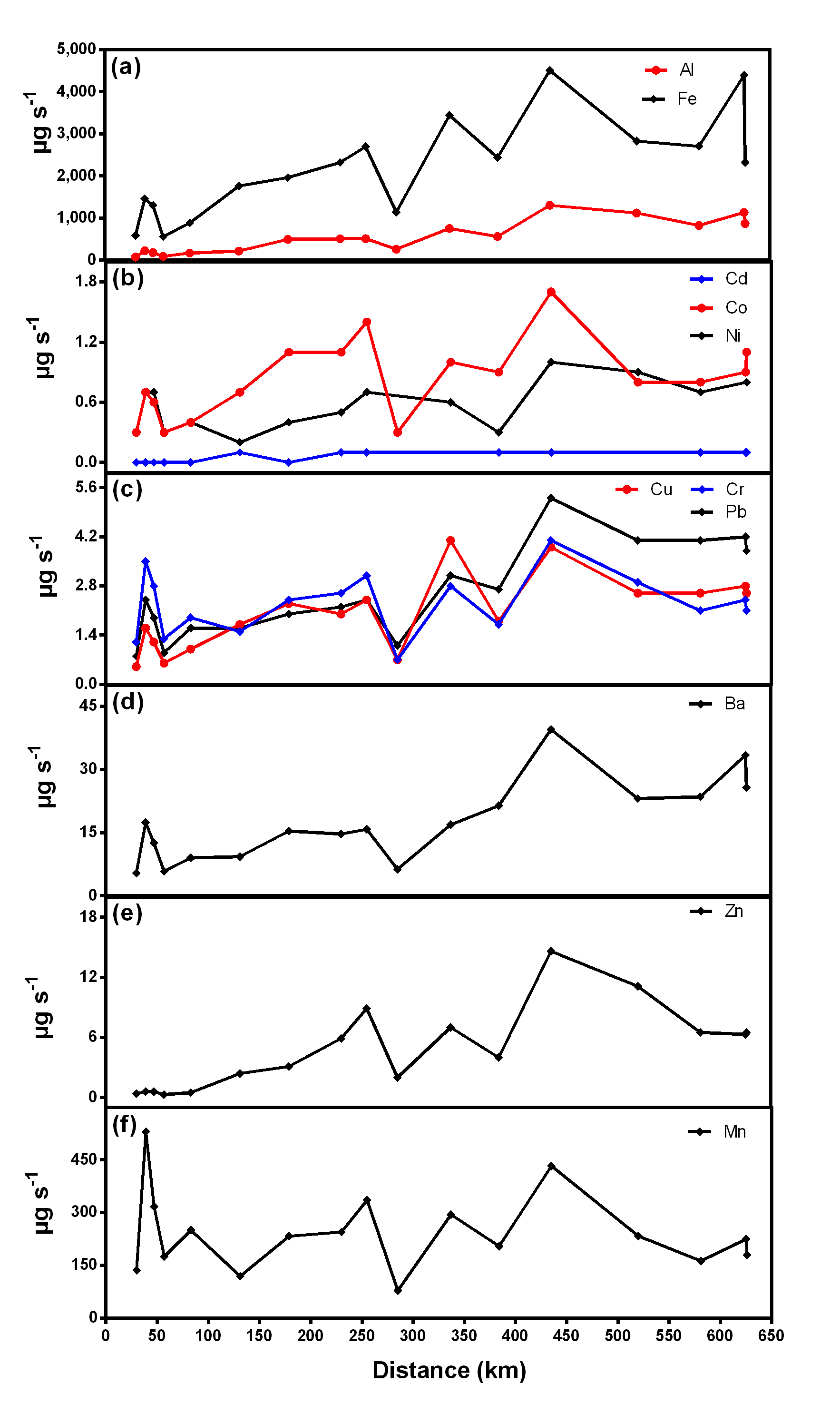


**Figure S3.** Fluxes of particulate trace metals along the Doce river downstream Samarco dam. Only the stations along the flow of the tailing slurry are presented.

**Figure S4.** (A) Empirical model of the dilution factor for the Doce River basin from the dam. (B) Mean and standard deviation of suspended particulate matter (SPM) concentrations along the Gualaxo do Norte and Doce Rivers (blue straight line). The blue dashed line is the best-fit function of the SPM (Eq. 2), and the red line is the modeled SPM based on the dilution factor (Eq. 1).

**Table S1.** Concentrations of dissolved trace elements (μg L^-1^) along Doce river basin and concentrations for the certified reference material SLRS-6.

| **Station**  **number** | **Km downstream** | **Ba** | **Pb** | **Th** | **U** | **Al** | **Cr** | **Mn** | **Fe** | **Co** | **Ni** | **Cu** | **As** |
| --- | --- | --- | --- | --- | --- | --- | --- | --- | --- | --- | --- | --- | --- |
| 1* | 15.8 | 26.9  (0.26) | 0.77  (0.01) | 0.003  (0.000001) | 0.002  (0.0000003) | 8.49  (0.24) | 1.27  (0.03) | 124  (0.80) | 27.3  (0.31) | 0.076  (0.0001) | 2.55  (0.031) | 0.51  (0.026) | 0.33  (0.004) |
| 2 | 9.9 | 24.5 | 0.19 | 0.003 | < 0.0001 | 9.11 | 0.13 | 105 | 31.6 | 0.068 | 0.58 | 0.26 | 0.22 |
| 3 | 3.5 | 12.9 | 0.19 | 0.004 | < 0.0001 | 6.45 | 0.10 | 117 | 34.3 | 0.884 | 1.45 | 0.30 | 0.17 |
| 5 | 30.0 | 170 | 1.83 | 0.002 | 0.105 | 11.8 | 1.50 | 175 | 8.89 | 0.097 | 0.66 | 0.37 | 0.47 |
| 6 | 38.6 | 168 | 0.15 | 0.002 | 0.119 | 6.10 | 0.026 | 218 | 3.42 | 0.072 | 0.34 | 0.16 | 0.42 |
| 7 | 46.9 | 157 | 0.60 | 0.001 | 0.043 | 8.17 | 0.071 | 264 | 8.42 | 0.124 | 0.36 | 0.32 | 0.39 |
| 8* | 81.8 | 11.4  (0.12) | 1.27  (0.03) | 0.012  (0.000001) | 0.006  (0.0000003) | 19.1  (0.46) | 0.28  (0.01) | 31.8  (0.14) | 51.2  (1.00) | 0.071  (0.003) | 1.00  (0.012) | 0.75  (0.208) | 1.73  (0.007) |
| 9 | 72.7 | 12.5 | 10.1 | 0.012 | 0.007 | 23.0 | 2.49 | 35.0 | 68.7 | 0.086 | 1.74 | 0.60 | 1.79 |
| 10 | 57.2 | 66.0 | 0.70 | 0.002 | 0.008 | 9.84 | 0.12 | 273 | 17.0 | 0.107 | 0.38 | 0.44 | 0.43 |
| 11 | 85.0 | 21.1 | 0.79 | 0.019 | 0.008 | 18.1 | 0.25 | 2.94 | 80.7 | 0.067 | 0.53 | 1.56 | 0.16 |
| 12 | 82.8 | 49.5 | 1.13 | 0.004 | 0.003 | 10.8 | 0.10 | 235 | 25.0 | 0.090 | 0.36 | 0.67 | 0.30 |
| 13* | 131 | 65.8  (0.53) | 1.99  (0.01) | 0.009  (0.000002) | 0.013  (0.0000002) | 42.7  (0.03) | 0.33  (0.01) | 110  (0.97) | 69.2  (0.51) | 0.164  (0.001) | 1.12  (0.004) | 1.80  (0.016) | 0.86  (0.012) |
| 14* | 179 | 32.4  (1.20) | 0.24  (0.01) | 0.006  (0.0008) | 0.002  (0.0000002) | 15.2  (0.47) | 0.39  (0.004) | 3.63  (0.09) | 40.3  (0.24) | 0.061  (0.001) | 0.62  (0.005) | 1.07  (0.019) | 0.30  (0.018) |
| 15 | 230 | 32.1 | 0.15 | 0.006 | 0.003 | 11.4 | 0.21 | 2.01 | 33.7 | 0.062 | 0.66 | 1.05 | 0.31 |
| 16 | 255 | 31.8 | 0.13 | 0.003 | 0.001 | 2.06 | 0.14 | 2.19 | 14.6 | 0.060 | 0.48 | 1.01 | 0.32 |
| 17 | 285 | 22.5 | 0.11 | 0.019 | 0.026 | 12.5 | 0.19 | 75.2 | 38.2 | 0.151 | 0.59 | 1.12 | 0.35 |
| 18* | 337 | 31.3  (0.23) | 0.16  (0.001) | 0.010  (0.000001) | 0.011  (0.000001) | 9.50  (0.40) | 0.16  (0.003) | 25.0  (0.04) | 33.8  (0.85) | 0.074  (0.001) | 0.60  (0.011) | 1.14  (0.024) | 0.43  (0.009) |
| 19 | 384 | 51.0 | 0.18 | 0.043 | 0.086 | 27.9 | 0.20 | 3.77 | 102 | 0.114 | 0.84 | 1.63 | 0.72 |
| 20 | 435 | 32.0 | 0.15 | 0.011 | 0.019 | 24.2 | 0.19 | 1.54 | 42.4 | 0.076 | 0.60 | 1.21 | 0.32 |
| 21* | 520 | 55.1  (0.29) | 0.16  (0.002) | 0.026  (0.002) | 0.021  (0.0016) | 27.3  (0.03) | 0.25  (0.01) | 45.1  (0.33) | 67.8  (0.13) | 0.105  (0.002) | 0.82  (0.009) | 1.49  (0.043) | 0.37  (0.006) |
| 22 | 581 | 39.9 | 0.79 | 0.017 | 0.016 | 17.2 | 0.17 | 1.63 | 43.4 | 0.057 | 0.49 | 1.26 | 0.26 |
| 23* | 625 | 42.5  (0.07) | 0.23  (0.01) | 0.016  (0.0008) | 0.014  (0.000006) | 20.7  (0.70) | 0.17  (0.01) | 1.74  (0.003) | 49.7  (1.53) | 0.058  (0.001) | 0.41  0.004 | 1.23  (0.064) | 0.23  (0.009) |
| 24 | 626 | 35.3 | 0.38 | 0.016 | 0.013 | 23.3 | 0.17 | 1.25 | 54.7 | 0.060 | 0.36 | 1.18 | 0.23 |
| 25 | 627 | 48.5 | 0.07 | 0.013 | 3.07 | 6.18 | 0.15 | 10.5 | 3.82 | 0.051 | 0.17 | 0.26 | 0.48 |
| 26* | 627 | 63.2  (0.26) | 0.27  (0.003) | 0.007  (0.000001) | 0.026  (0.000003) | 9.33  (0.002) | 0.24  (0.003) | (0.04) | 14.6  (0.29) | 0.060  (0.022) | 0.33  (0.0004) | 0.62  (0.001) | 0.21  (0.009) |
| 27 | 629 | 53.1 | 0.52 | 0.011 | 1.38 | 6.00 | 0.10 | 4.04 | 2.95 | 0.031 | 0.18 | 0.32 | 0.40 |
| 28 | 632 | 12.4 | 6.48 | 0.009 | 5.25 | 2.82 | 0.14 | 0.91 | 1.15 | 0.013 | 0.11 | 0.01 | 1.01 |
| SLRS -6* | - | 14.0  (0.16) | 0.19  (0.01) | 0.03  (0.002) | 0.07  (0.003) | 33.2  (0.60) | 0.25 (0.01) | 2.47  (0.05) | 80.7  (1.74) | 0.06  (0.002) | 0.62 (0.01) | 24.8  (0.36) | 0.59  (0.01) |
| % Recovery |  | 98.2 | 111 | - | 95.6 | 97.9 | 97.9 | 116 | 95.4 | 106 | 101 | 103 | 103 |

*Values represent mean (1 std).

**Table S2.** Mean and standard deviation of particulate metal concentrations (μg g^-1^) along Doce river basin.

| Station  number | Km downstream | Al | As | Ba | Cd | Co | Cr | Cu | Fe | Ni | Mn | Pb | Zn |
| --- | --- | --- | --- | --- | --- | --- | --- | --- | --- | --- | --- | --- | --- |
| 1 | 15.8 | 2843  (682) | <0.35 | 457  (8.35) | <0.03 | 6.98  (0.22) | 34.6  (3.33) | 11.1  (0.49) | 7697  (316) | 9.60  (0.19) | 5376  (131) | 8.79  (4.34) | 66  (32.4) |
| 2 | 9.9 | 2360  (54.2) | <0.35 | 287  (13.2) | 0.276 | 4.70  (0.18) | 57.5  (72.2) | 11.8  (0.43) | 10105  (44.8) | 2.25 | 6879  (321) | 20.0 | 71  (27.7) |
| 3 | 3.5 | 3361  (516) | <0.35 | 57.9  (5.28) | <0.03 | 2.64 | 24.9  (5.23) | 14.7  (1.20) | 27699  (5030) | 9.02  (3.33) | 768  (13.8) | 12.5  (2.82) | 229  (28.6) |
| 5 | 30.0 | 494 (2.48) | <0.35 | 34.7  (0.49) | 0.08  (0.003) | 1.72  (0.03) | 7.42  (0.11) | 3.36  (0.00003) | 3789  (70.3) | 1.87  (0.05) | 882  (20.3) | 5.40  (0.21) | 3  (0.89) |
| 6 | 38.6 | 463  (3.01) | <0.35 | 35.1  (0.14) | 0.07  (0.01) | 1.46  (0.01) | 7.11  (0.10) | 3.22  (0.02) | 2945  (18.4) | 1.49  (0.06) | 1070  (10.3) | 4.80  (0.01) | 1  (0.04) |
| 7 | 46.9 | 458 (14.7) | <0.35 | 31.8  (0.29) | 0.08  (0.004) | 1.60  (0.002) | 6.96  (0.05) | 3.12  (0.03) | 3289  (110) | 1.86  (0.23) | 799  (20.4) | 4.89  (0.23) | 2  (0.05) |
| 8 | 81.8 | 10652 | 286  (191) | 883  (465) | 2.41  (1.53) | 75.5  (42.4) | 84.9  (57.0) | 62.3  (32.68) | 48143 | 57.1  (32.2) | 15514  (6379) | 48.0  (24.3) | 223  (119) |
| 9 | 72.7 | 4984 (456) | 78.0  (3.57) | 287  (18.5) | 0.43  (0.10) | 20.1  (1.20) | 19.7  (1.47) | 20.4  (1.08) | 18681  (1353) | 12.3  (0.42) | 6420  (273) | 13.4  (0.02) | 33  (1.09) |
| 10 | 57.2 | 635  (20.7) | <0.35 | 40.0  (1.56) | 0.12  (0.02) | 2.04  (0.02) | 9.25  (0.18) | 4.25  (0.06) | 3885  (383) | 1.94  (0.07) | 1213  (10.3) | 5.93  (0.08) | 2  (0.04) |
| 11 | 85.0 | 9668 | <0.35 | 192  (21.0) | 1.05  (0.01) | 19.0  (2.19) | 32.1  (5.38) | 38.1  (4.56) | 40131  (5184) | 6.49  (1.60) | 1808  (228) | 29.6  (5.50) | 61  (2.07) |
| 12 | 82.8 | 754 (27.3) | <0.35 | 39.2  (0.27) | 0.17  (0.07) | 1.65  (0.09) | 8.42  (0.03) | 4.43  (0.002) | 3848  (408) | 1.52  (0.03) | 1082  (7.39) | 6.99  (0.21) | 2  (0.20) |
| 13 | 131 | 1993 (261) | <0.35 | 84.3  (35.7) | 0.59  (0.24) | 6.73  (5.53) | 13.8  (5.30) | 15.1  (9.80) | 15950  (9646) | 2.10  (1.11) | 1075  (54.3) | 14.4  (5.44) | 21  (18.4) |
| 14 | 179 | 2228 (9.42) | <0.35 | 68.5  (0.50) | 0.21  (0.04) | 4.83  (0.27) | 10.5  (0.47) | 10.0  (0.11) | 8757  (188) | 1.94  (0.16) | 1038  (9.13) | 8.83  (0.52) | 14  (3.30) |
| 15 | 230 | 1918 (547) | <0.35 | 55.4  (0.12) | 0.28  (0.05) | 4.12  (0.18) | 9.93  (0.16) | 7.67  (0.09) | 8745  (1065) | 2.07  (0.20) | 920  (9.29) | 8.37  (0.93) | 22  (5.69) |
| 16 | 255 | 1725 (92.3) | <0.35 | 52.8  (13.3) | 0.20  (0.04) | 4.62  (0.35) | 10.4  (0.76) | 8.17  (0.28) | 9028  (307) | 2.40  (0.46) | 1124  (45.7) | 8.16  (0.62) | 30  (5.68) |
| 17 | 285 | 3826 (137) | <0.35 | 90.3  (10.4) | <0.03 | 3.78  (1.13) | 10.5  (0.01) | 9.83  (1.10) | 16491  (820) | <0.13 | 1126  (129) | 16.2  (0.97) | 29  (0.44) |
| 18 | 337 | 3309 (327) | <0.35 | 74.3  (2.18) | <0.03 | 4.35  (0.21) | 12.4  (0.08) | 17.9  (9.23) | 15104  (1269) | 2.79 | 1291  (34.3) | 13.7  (0.95) | 31 |
| 19 | 384 | 2504 (397) | <0.35 | 94.6  (2.94) | 0.28  (0.08) | 4.11  (0.40) | 7.38  (0.39) | 7.87  (0.21) | 10791  (839) | 1.27  (0.42) | 906  (18.2) | 11.8  (1.31) | 18  (0.07) |
| 20 | 435 | 2769 (918) | <0.35 | 83.7  (14.6) | 0.29  (0.10) | 3.64  (0.97) | 8.66  (2.68) | 8.19  (1.31) | 9555  (1544) | 2.17  (0.49) | 916  (128) | 11.3  (1.67) | 31  (9.71) |
| 21 | 520 | 4648 (606) | <0.35 | 95.6  (15.5) | <0.03 | 3.31  (1.04) | 11.8  (0.46) | 11.0  (1.67) | 11724  (1556) | 3.68  (1.58) | 967  (166) | 17.0  (2.04) | 46  (4.55) |
| 22 | 581 | 4778 (892) | <0.35 | 136  (24.8) | 0.82  (0.14) | 4.72  (0.97) | 12.1  (3.06) | 15.0  (2.04) | 15676  (1102) | 3.91  (1.46) | 941  (225) | 23.4  (0.46) | 37  (8.65) |
| 23 | 625 | 4028 (75.3) | <0.35 | 118  (7.68) | 0.37  (0.04) | 3.21  (0.20) | 8.50  (0.16) | 9.80  (0.59) | 15563  (166) | <0.13 | 795  (46.5) | 14.8  (0.03) | 22  (0.66) |
| 24 | 626 | 4324 (337) | <0.35 | 127  (13.1) | 0.62  (0.09) | 5.28  (0.60) | 10.2  (1.08) | 12.9  (1.52) | 11516  (2066) | 3.97  (0.82) | 889  (91.2) | 19.1  (3.57) | 32  (3.65) |
| 25 | 627 | 5936 (250) | <0.35 | 35.2  (4.33) | 0.41  (0.07) | 3.76  (0.92) | 10.4  (1.02) | 12.6  (1.20) | 20768  (310) | 4.08  (0.10) | 775  (109) | 18.0  (2.28) | 34  (9.09) |
| 26 | 627 | 4094 (794) | <0.35 | 43.4  (8.87) | 0.54  (0.06) | 4.27  (0.44) | 9.16  (2.03) | 10.9  (2.03) | 13197  (2627) | 3.18  (0.85) | 684  (156) | 15.6  (4.64) | 23  (3.81) |
| 27 | 629 | 3597 (77.4) | <0.35 | 26.0  (1.05) | <0.03 | 0.86 | 7.89  (0.63) | 9.66  (0.66) | 12807  (576) | <0.13 | 409  (19.2) | 15.7  (3.67) | 27  (1.82) |
| 28 | 632 | 8389 (601) | 15.2  (1.00) | 16.4  (0.66) | <0.03 | 3.20  (0.64) | 23.3  (0.60) | 3.25  (0.84) | 13765  (4285) | 4.54  (0.02) | 674  (75.6) | 30.1  (21.9) | 34  (0.54) |

**Table S3.** Mean (standard deviation) concentrations (μg g^-1^) for the pseudo-total (PT) and bioavailable (B) fraction of metals in sediments along Doce river basin and for certified reference material MESS-3.

| **Station**  **number** | **Fraction** | **Al** | **As** | **Ba** | **Cd** | **Co** | **Cr** | **Cu** | **Fe** | **Ni** | **Mn** | **Pb** | **Hg*** | **Zn** |
| --- | --- | --- | --- | --- | --- | --- | --- | --- | --- | --- | --- | --- | --- | --- |
| 1 | PT | 28157  (491) | 34.4  (0.93) | 167  (3.38) | 11.3  (0.52) | 6.75  (0.99) | 184  (130) | 14.8  (2.22) | 227600  (8054) | 105  (100) | 4198  (120) | 19.3  (1.47) | 85.1  (2.22) | 37.0  (0.17) |
|  | B | 1876  (28.0) | <0.63 | 43.1  (0.66) | 0.26  (0.00) | 1.40  (0.06) | 2.97  (0.02) | 0.76  (0.02) | 2559  (66.9) | <0.28 | 1425  (19.4) | 2.32  0.20 |  | 0.31  (0.03) |
| 2 | PT | 10701  (603) | 40.4  (1.13) | 298  (1.19) | 8.05  (0.23) | 15.7  (0.93) | 66.7  (27.9) | 27.1  (2.30) | 155902  (8955) | 45.8  (25.1) | 5022  (307) | 12.3  (0.78) | 478 | 33.9  (2.45) |
|  | B | 1166 (14.5) | <0.63 | 61.3  (0.40) | 0.27  (0.00) | 12.3  (0.19) | 2.66  (0.05) | 3.19  (0.11) | 2048  (7.27) | 1.44  (0.05) | 1523  (24.6) | 2.09  (0.81) |  | 1.90  (0.04) |
| 3 | PT | 8796  (222) | <0.97 | 40.7  (0.27) | 7.89  (0.21) | 23.3  (2.98) | 45.6  (21.4) | 10.1  (0.53) | 164410  (11745) | 27.6  (17.1) | 317  (14.9) | 13.3  (0.14) | 33.7 | 30.7  (1.41) |
|  | B | 1757 (0.54) | <0.63 | 9.93  (0.13) | 0.15  (0.01) | 25.6  (1.45) | 3.14  (0.05) | 1.92  (0.01) | 1889  (74.0) | 1.06  (0.20) | 253  (2.18) | 3.01  (0.04) |  | 1.42  (0.07) |
| 4 | PT | 2617  (169) | <0.97 | 24.8  (0.06) | 8.35  (0.03) | 8.51  (1.14) | 21.8  (6.36) | 4.50  (0.13) | 170893  (912) | 12.3 | 258  (2.98) | 9.71  (1.17) | 15.4 | 26.3  (0.86) |
|  | B | 250 (6.80) | <0.63 | 5.59  (0.30) | 0.10  (0.01) | 5.6  (0.10) | 1.04  (0.05) | 0.12  (0.01) | 921  (4.81) | <0.28 | 159  (6.62) | <0.28 |  | <0.21 |
| 5 | PT | 1282  (38.8) | <0.97 | 111  (11.0) | 14.7  (0.27) | 14.0  (2.32) | 16.3  (0.84) | 3.29  (0.20) | 249087  (8458) | 8.93  (1.66) | 769  (34.4) | 5.65  (0.75) | 23.5 | 39.8  (0.22) |
|  | B | 127 (4.13) | <0.63 | 25.8  (0.49) | 0.09  (0.01) | 11.8  (0.49) | 0.62  (0.03) | <0.28 | 866  (60.5) | <0.28 | 224  (2.66) | <0.28 |  | <0.21 |
| 6 | PT | 1282 (24.7) | <0.97 | 15.0  (2.06) | 6.55  (0.14) | 19.9  (0.11) | 17.6  (14.8) | 2.61  (0.96) | 169768  (664) | 4.32  (3.94) | 168  (5.92) | 8.80  (0.34) | 7.81 | 22.8  (0.76) |
|  | B | 144 (10.6) | <0.63 | 6.31  (0.45) | 0.09  (0.01) | 18.6  (0.71) | 0.42  (0.01) | <0.28 | 649  (9.35) | <0.28 | 101  (2.53) | <0.28 |  | <0.21 |
| 7 | PT | 2125 (35.5) | <0.97 | 15.9  (0.05) | 7.67  (0.16) | 19.2  (0.82) | 7.96  (1.20) | 1.65  (0.07) | 171014  (5158) | 2.42  (0.05) | 176  (4.18) | 11.1  (1.05) | 18.6 | 21.9  (0.17) |
|  | B | 147 (5.97) | <0.63 | 6.82  (0.15) | 0.10  (0.00) | 17.2  (0.97) | 1.00  (0.05) | <0.28 | 773  (61.2) | <0.28 | 129  (0.15) | <0.28 |  | <0.21 |
| 8 | PT | 19347 (288) | 87.7  (5.68) | 90.9  (0.51) | 3.97  (0.01) | 18.9 (0.71) | 74.8  (1.68) | 15.09  (0.32) | 87467  (850) | 28.4  (0.57) | 1262  (65.6) | 9.23  (0.11) | 23.7 | 34.9  (0.31) |
|  | B | 1183 (19.0) | 6.90  (0.56) | 43.3  (1.44) | 0.22  (0.00) | 12.6  (0.92) | 4.00  (0.13) | 2.80  (0.08) | 1760  (21.1) | 3.48  (0.17) | 972  (2.62) | 1.00  (0.07) |  | 4.31  (0.5) |
| 9 | PT | 8822 (622) | 97.7  (11.3) | 63.5  (2.28) | 5.11  (0.24) | 37.2  (2.15) | 63.42  (1.32) | 10.14  (0.01) | 116948  (6701) | 18.5  (0.94) | 1170  (40.2) | 7.59  (0.74) | 13.3 | 30.4  (1.42) |
|  | B | 816 (18.2) | 5.79  (0.06) | 32.5  (0.06) | 0.11  (0.01) | 27.2  (0.88) | 3.00  (0.13) | 1.25  (0.09) | 1076  (28.1) | 2.40  (0.11) | 687  (24.9) | <0.28 |  | 2.27  (0.10) |
| 10 | PT | 1471 (90.7) | <0.97 | 16.0  (0.39) | 11.2  (0.06) | 21.6  (0.61) | 20.1  (1.32) | 1.75  (0.05) | 286175  (2882) | 8.00  (7.29) | 172  (5.65) | 13.1  (0.07) | 13.4 | 34.8  (1.93) |
|  | B | 122 (1.25) | <0.63 | 6.06  (0.20) | 0.10  (0.01) | 20.6  (1.48) | 0.65  (0.02) | <0.28 | 706  (28.4) | <0.28 | 111  (2.92) | <0.28 |  | <0.21 |
| 11 | PT | 77824 (249) | 40.5  (0.38) | 146  (1.29) | 2.60  (0.00) | 18.6  (0.39) | 94.1 | 32.9  (0.30) | 44090  (1353) | 45.8 | 707  (7.85) | 12.0  (1.50) | 34.1 | 82.4  (0.52) |
|  | B | 3056 (58.8) | <0.63 | 74.3  (0.45) | 1.10  (0.04) | 13.1  (0.67) | 9.79  (0.64) | 10.9  (0.10) | 9482  (169) | 4.99  (0.12) | 533  (3.33) | 9.20  (0.24) |  | 30.26  (0.53) |
| 12 | PT | 3367 (89.8) | <0.97 | 17.9  (0.47) | 8.80  (0.11) | 23.3 (3.58) | 13.3  (0.60) | 2.83  (0.12) | 162554  (1381) | 3.69  (0.05) | 215  (30.1) | 10.4  (0.96) | 12.5 | 25.2  (0.31) |
|  | B | 299 (9.60) | <0.63 | 7.87  (0.15) | 0.10  (0.01) | 27.0  (1.79) | 1.34  (0.03) | 0.76  (0.04) | 933  (11.9) | 0.41 | 145  (9.43) | 0.72  (0.05) |  | 0.34  (0.03) |
| 13 | PT | 10033  (667) | <0.97 | 43.2  (1.10) | 8.89  (0.17) | 10.1  (0.50) | 32.2  (6.07) | 6.74  (0.34) | 152411  (688) | 11.2  (3.83) | 414  (9.61) | 9.68  (0.32) | 24.4 | 33.9  (0.57) |
|  | B | 797  (28.3) | <0.63 | 22.6  (0.77) | 0.29  (0.01) | 8.40  (0.46) | 3.06  (0.06) | 1.67  (0.10) | 2258  (45.6) | 1.15  (0.03) | 339  (2.65) | 1.10  (0.22) |  | 3.95  (0.06) |
| 14 | PT | 18280  (126) | <0.97 | 66.6  (0.48) | 6.99  (0.30) | 12.6  (0.88) | 41.2  (5.90) | 10.8  (0.11) | 120009  (288) | 16.7  (3.11) | 248  (1.04) | 8.72  (1.15) | 34.8 | 36.1  (0.15) |
|  | B | 1142 (4.11) | <0.63 | 37.1  (0.34) | 0.39  (0.03) | 11.2  (0.79) | 4.77  (0.24) | 3.09  (0.05) | 2907  (173) | 2.44  (0.10) | 435  (4.80) | 2.45  (0.10) |  | 6.00  (0.05) |
| 15 | PT | 32834  (1048) | <0.97 | 98.0  (0.34) | 6.23  (0.13) | 12.5  (0.23) | 53.7  (1.03) | 15.9  (0.15) | 103983  (1509) | 22.7  (1.06) | 346  (0.38) | 11.2  (0.56) | 49.4 | 49.7  (0.65) |
|  | B | 1980 (9.99) | <0.63 | 52.5  (0.28) | 0.62  (0.04) | 11.4  (0.36) | 9.51  (0.05) | 5.37  (0.08) | 4659  (204) | 4.70  (0.13) | 594  (1.01) | 5.89  (0.27) |  | 15.3  (0.08) |
| 16 | PT | 20352  (433) | <0.97 | 37.0  (0.77) | 8.05  (0.11) | 7.63  (0.37) | 42.8  (4.83) | 10.6  (0.18) | 130155  (2583) | 15.9  2.27 | 495  (14.8) | 10.1  (0.05) | 33.1 | 41.8  (1.21) |
|  | B | 1308 (24.7) | <0.63 | 33.2  (0.32) | 0.45  (0.02) | 6.60  (0.14) | 6.05  (0.26) | 3.30  (0.14) | 3140  (194) | 3.01  (0.20) | 435  (3.98) | 2.45  (0.12) |  | 9.84  (0.25) |
| 17 | PT | 34868  (0.50) | <0.97 | 91.4  (1.94) | 8.57  (0.15) | 9.24  (0.17) | 48.9  (0.34) | 15.2  (0.08) | 138151  (493) | 20.1  (0.07) | 696  (1.0) | 12.7  (0.39) | 55.7 | 51.8  (0.52) |
|  | B | 1541 (23.6) | <0.63 | 44.8  (0.56) | 0.60  (0.02) | 8.10  (0.24) | 7.40  (0.09) | 4.56  (0.10) | 4790  (13.7) | 3.43  (0.09) | 626  (2.6) | 5.25  (0.23) |  | 11.51  (0.16) |
| 18 | PT | 52531 (451) | <0.97 | 129  (0.11) | 7.64  (0.12) | 11.4  (0.37) | 60.0  (0.64) | 20.3  (0.10) | 120387  (382) | 25.2  (0.30) | 820  (3.58) | 14.7  (0.20) | 65.3 | 59.6  (0.05) |
|  | B | 2236 (4.03) | <0.63 | 65.2  (0.64) | 0.86  (0.02) | 9.50  (0.29) | 9.31  (0.19) | 7.02  (0.16) | 6792  (83.3) | 4.30  (0.14) | 770  (9.57) | 8.27  (0.37) |  | 16.01  (0.17) |
| 19 | PT | 76945 (567) | <0.97 | 185  (0.97) | 6.49  (0.39) | 13.3  (0.70) | 67.1  (1.76) | 25.3  (0.13) | 99906  (1019) | 28.1  (0.19) | 1271  (24.1) | 19.6  (0.85) | 76.7 | 74.5  (0.67) |
|  | B | 2940 (232) | <0.63 | 106  (1.49) | 1.17  (0.03) | 8.90  (0.23) | 9.00  (0.59) | 9.65  (0.16) | 9014  (355) | 4.32  (0.01) | 1125  (50) | 13.9  (0.41) |  | 18.75  (0.12) |
| 20 | PT | 48840 (180) | <0.97 | 139  (0.42) | 6.65  (0.03) | 10.6 (0.02) | 54.7  (0.42) | 19.3  (0.50) | 99346  (646) | 22.5  (0.04) | 1009  (13.7) | 13.2  (1.10) | 64.5  (3.38) | 56.8  (0.59) |
|  | B | 2329 (57.8) | <0.63 | 82.4  (0.65) | 0.90  (0.05) | 7.90  (0.47) | 9.00  (0.15) | 6.69  (0.05) | 7994  (104) | 3.91  (0.05) | 1059  (11.8) | 8.75  (0.12) |  | 15.0  (0.01) |
| 21 | PT | 64008 (752) | <0.97 | 182  (2.36) | 5.57  (0.02) | 11.7  (0.05) | 60.9  (0.21) | 22.1  (0.12) | 83028  (789) | 25.3  (0.22) | 867  (1.80) | 14.5  (0.00) | 60.1 | 68.8  (2.08) |
|  | B | 3188 (83.9) | <0.63 | 109  (1.25) | 0.95  (0.05) | 8.50  (0.19) | 8.84  (0.24) | 8.09  (0.06) | 8958  (433) | 4.46  (0.18) | 925  (3.78) | 10.2  (0.52) |  | 18.5  (0.43) |
| 22 | PT | 6380 (220) | <0.97 | 26.5  (2.62) | 4.12  (0.09) | 19.4  (0.01) | 22.2  (4.10) | 6.09  (0.23) | 47626  (1406) | 5.98  (0.65) | 360  (3.27) | 4.22  (0.15) | 22.8 | 24.3  (0.66) |
|  | B | 790 (71.6) | <0.63 | 13.4  (0.83) | 0.10  (0.00) | 19.4  (0.91) | 1.28  (0.12) | 0.36  (0.05) | 1069  (88.7) | 0.52  (0.05) | 75.8  (4.84) | 1.56  (0.10) |  | 2.10  (0.15) |
| 23 | PT | 9400 (848) | <0.97 | 25.0  (0.14) | 3.06  (0.00) | 23.3  (0.17) | 21.9  0.25 | 4.02  (0.22) | 37462  (1204) | 5.92  0.27 | 271  (8.01) | 2.67 | 13.2 | 21.2  (0.09) |
|  | B | 916 (14.1) | <0.63 | 14.1  (0.38) | <0.07 | 21.0  (1.64) | 1.63  (0.06) | 1.07  (0.07) | 1318  (52.4) | 0.62  (0.05) | 78.8  (4.20) | 1.64  (0.12) |  | 2.43  (0.09) |
| 24 | PT | 92837 (670) | <0.97 | 88.9  (0.55) | 4.69  (0.04) | 12.9 (0.00) | 66.3  (0.98) | 18.0  (0.20) | 68597  (20.6) | 26.8  (0.54) | 1443  (26.2) | 12.0  (0.56) | 36.3 | 60.9  (1.67) |
|  | B | 3893 (280) | <0.63 | 23.3  (0.58) | 0.24  (0.00) | 9.30  (0.23) | 11.0  (0.46) | 6.15  (0.02) | 11181  (641) | 3.36  (0.14) | 1349  (8.84) | 12.0  (0.17) |  | 14.6  (0.32) |
| 25 | PT | 94045 (4591) | <0.97 | 117  (6.14) | 12.5  (0.55) | 12.0  (0.11) | 77.2  (2.88) | 26.7  (1.63) | 182419  (8126) | 34.4  (1.96) | 804  (25.6) | 18.5  (1.38) | 69.6 | 78.6  (2.69) |
|  | B | 3467 (60.4) | <0.63 | 34.3  (0.33) | 0.21  (0.00) | 7.20  (0.10) | 11.4  (0.19) | 10.4  (0.23) | 10677  (161) | 3.88  (0.05) | 775  (15.6) | 13.9  (0.33) |  | 17.0  (0.27) |
| 26 | PT | 6915 (257) | <0.97 | 20.0  (0.34) | 1.71  (0.01) | 20.2  (0.87) | 13.9  (0.33) | 3.29  (0.01) | 24901  (497) | 4.57  (0.23) | 163  (2.13) | 2.69 | 3.57 | 13.0  (0.11) |
|  | B | 700 (9.04) | <0.63 | 5.47  (0.07) | <0.07 | 26.3  (0.20) | 1.53  (0.04) | 0.26  (0.01) | 1132  (19.1) | 0.34  (0.02) | 75.2  (1.77) | 1.38  (0.08) |  | 1.72  (0.04) |
| 27 | PT | 3281 (7.91) | <0.97 | 8.20  (0.26) | 0.42  (0.00) | 25.0  (0.19) | 6.12  0.04 | 0.81  (0.05) | 5541  (85.9) | 1.77  0.06 | 91.6  (1.18) | <0.43 | 3.36 | 6.40  (0.02) |
|  | B | 325 (6.11) | <0.63 | 2.57  (0.06) | <0.07 | 31.6  (1.89) | 0.75  (0.06) | <0.28 | 411  (10.6) | <0.28 | 25.1  (1.12) | 1.05 |  | 0.60  (0.04) |
| 28 | PT | 15056 (443) | <0.97 | 34.1  (1.67) | 7.16  (0.03) | 19.9 (0.81) | 43.3  (1.50) | 8.50  (0.08) | 95185  (1556) | 12.1  (1.01) | 418  (1.17) | 8.37  (1.52) | 7.68 | 43.2  (0.05) |
|  | B | 1031 (80.8) | <0.63 | 8.09  (0.13) | 0.29  (0.02) | 18.8  (1.64) | 2.89  (0.19) | 0.30  (0.05) | 2406  (142) | 1.41  (0.06) | 152  (1.48) | 3.50  (0.24) |  | 4.58  (0.10) |
| MESS-3 | PT | 6.79%  (0.10) | 19.3  (1.4) | 563  (8.7) |  | 8.98  (0.38) | 62.1  (1.6) | 24.8  (0.93) | 3.58%  (0.11) | 30.2  (1.0) | 232  (4.57) | 9.33  (0.29) |  | 116  (1.98) |
|  | B | 0.34%  (0.01) | 5.69  (0.34) | 141  (12.0) |  | 4.01  (0.14) | 4.87  (0.25) | 15.5  (0.31) | 1.39%  (0.04) | 9.12  (0.25) | 147  (8.75) | 9.88  (0.20) |  | 57.5  (1.56) |

Hg* = ng g^-1^.

**Table S4.** Loadings for Principal Component Analysis (Figure 6). Values in red identify the variables of each factor.

|  | Variables | PC 1 | PC 2 |
| --- | --- | --- | --- |
|  | SPM (mg/L) | 0.51423 | 0.480705 |
| Particulate | Fe | -0.63724 | -0.489047 |
|  | Al | -0.64825 | -0.438412 |
|  | As | -0.21885 | -0.812158 |
|  | Ba | -0.64115 | -0.520595 |
|  | Cd | -0.42667 | -0.630064 |
|  | Co | -0.65138 | -0.655862 |
|  | Cr | -0.53281 | -0.624855 |
|  | Cu | -0.66483 | -0.552025 |
|  | Mn | -0.45768 | -0.612532 |
|  | Ni | -0.47596 | -0.615774 |
|  | Pb | -0.54584 | -0.523379 |
|  | Zn | -0.71682 | -0.402175 |
| Dissolved | Ba | 0.63041 | 0.470607 |
|  | Pb | 0.09902 | -0.608953 |
|  | Th | -0.49034 | 0.081663 |
|  | U | 0.10159 | -0.021409 |
|  | Al | -0.35756 | -0.111823 |
|  | Cr | -0.13211 | -0.378918 |
|  | Mn | 0.29655 | -0.016993 |
|  | Fe | -0.60221 | -0.086573 |
|  | Co | -0.03276 | 0.015918 |
|  | Ni | -0.43727 | -0.291363 |
|  | Cu | -0.49124 | 0.196254 |
|  | As | -0.15337 | -0.623929 |
| Pseudo Total fraction | Fe | -0.14244 | 0.298653 |
|  | Al | -0.90016 | 0.225342 |
|  | As | -0.44074 | -0.700523 |
|  | Ba | -0.87165 | 0.194014 |
|  | Cd | -0.13960 | 0.425985 |
|  | Co | 0.48186 | -0.589194 |
|  | Cr | -0.92988 | -0.011694 |
|  | Cu | -0.96405 | 0.141492 |
|  | Mn | -0.79948 | -0.032198 |
|  | Ni | -0.90860 | 0.049417 |
|  | Pb | -0.60677 | 0.403131 |
|  | Zn | -0.77265 | 0.414178 |
|  | Hg | -0.71044 | 0.346633 |
| Bioavailable Fraction | Fe | -0.85255 | 0.437647 |
|  | Al | -0.89716 | 0.133165 |
|  | As | -0.24327 | -0.813483 |
|  | Ba | -0.87578 | 0.215662 |
|  | Cd | -0.70427 | 0.424379 |
|  | Co | 0.61929 | -0.394205 |
|  | Cr | -0.87420 | 0.336268 |
|  | Cu | -0.84039 | 0.359208 |
|  | Mn | -0.88738 | 0.144790 |
|  | Ni | -0.84192 | 0.181840 |
|  | Pb | -0.74373 | 0.477423 |
|  | Zn | -0.78128 | 0.314082 |
| Granulometry | Sand | 0.45644 | -0.300496 |
|  | Silt | -0.61287 | 0.395176 |
|  | Clay | -0.57166 | 0.116993 |

**Table S5**. HR ICP-MS characteristics and settings.

| Parameters |  |
| --- | --- |
| Sample cone | Nickel |
| Skimmer cone  Cyclonic twin bar spray chamber | Nickel  Quartz |
| RF power (W) | 1270 |
| Sample gas (Ar) flow rate (L min^-1^) | 1.03 |
| Auxiliary gas rate flow rate (L min^-1^) | 0.58 |
| Cool gas flow rate (L min^-1^) | 16.0 |
| Sample uptake rate (mL min^-1^) | 1.0 |

**Table S6.** ICP OES characteristics and settings.

| Parameters |  |
| --- | --- |
| Power (W) | 1300 |
| Plasma flow (L/min) | 15.0 |
| Auxiliary flow (L/min) | 1.5 |
| Nebulizer pressure (kPa) | 220 |
| Nebulizer gas flow (L/min) | 1.5 |
| Spray chamber | Sturman Master |
| Replicate read time (s) | 40.0 |
| Stabilization delay (s) | 15 |

**Table S7.** Distance of the sampling stations along the Gualaxo do Norte and Doce rivers from the dam (Figure 1); measured suspended particulate matter concentrations (SPM) with respective standard deviation; dilution factor (D); estimated river discharge (Q, m^3^s^-1^) at the station sites; estimated SPM_Fit_ from Eq. (2); modeled SPM_Mod_ with the dilution factor; and the ratio between modeled and estimated SPM values.

| Station number | Distance  (Km) | SPM  (mg L^-1^) | D(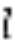) | Q  (m^3^s^-1^) | SPM_Fit_  (mg L^-1^) | SPM_mod_  (mg L^-1^l) | Mod/Fit |
| --- | --- | --- | --- | --- | --- | --- | --- |
| 5 | 32 | 14793 ± 1141 | 2.6 | 10 | 27083 | 21754 | 0.80 |
| 6 | 41 | 33087 ± 3606 | 3.7 | 15 | 17912 | 15231 | 0.85 |
| 7 | 50 | 19762 ± 3.4 | 5.0 | 20 | 12565 | 11369 | 0.90 |
| 10 | 61 | 5280 ± 313 | 6.8 | 27 | 8544 | 8343 | 0.98 |
| 12 | 88 | 4614 ± 105 | 12.5 | 50 | 4000 | 4558 | 1.14 |
| 13 | 139 | 1020 ± 105 | 27.1 | 108 | 1633 | 2103 | 1.29 |
| 14 | 190 | 1212 ± 862 | 46.3 | 185 | 975 | 1232 | 1.26 |
| 15 | 244 | 944 ± 9.2 | 70.4 | 282 | 696 | 810 | 1.16 |
| 16 | 270 | 896 ± 76.3 | 83.3 | 333 | 616 | 684 | 1.11 |
| 17 | 302 | 174 ± 22.3 | 99.3 | 397 | 544 | 574 | 1.06 |
| 18 | 357 | 443 ± 21.9 | 128.6 | 514 | 453 | 443 | 0.98 |
| 19 | 407 | 363 ± 29.6 | 155.5 | 622 | 393 | 367 | 0.93 |
| 20 | 461 | 639 ± 30.5 | 184.5 | 738 | 341 | 309 | 0.91 |
| 21 | 551 | 265 ± 44.0 | 228.1 | 912 | 273 | 250 | 0.92 |
| 22 | 616 | 170 ± 5.4 | 253.9 | 1016 | 235 | 224 | 0.95 |
| 23 | 662 | 263 ± 23.3 | 268.1 | 1072 | 211 | 213 | 1.01 |
| 24 | 664 | 188 ± 13.8 | 268.4 | 1073 | 211 | 212 | 1.00 |

**Table S8.** Gauge stations period of operation (TO), operation interval (OI), number of samples (NS, as monthly means), mean discharge (Q), Pearson correlation coefficient (R) and P value, number of synoptic samples for *Fazenda Ocidente* (NS_B_), mean discharge of the synoptic period for *Fazenda Ocidente* station (Q_S_), mean discharge of *Fazenda Ocidente* synoptic with each other station (Q_B_), and the ratio between Q_S_ to the Q_S_ at *Bicas* (Q_S-A_).

| **Stations** | | **TO** | **OI**  **(years)** | **NS** | **Q**  **(m^3^ s^-1^)** | **R(P)** | **NS_B_** | **Q_S_**  **(m^3^ s^-1^)** | **Q_B-Synoptic_**  **(m^3^ s^-1^)** | **Q_S_/Q_S-A_** |
| --- | --- | --- | --- | --- | --- | --- | --- | --- | --- | --- |
| A | ***Bicas*** | 1040-1966 | 27 | 299 | 4.0 | 0.91 (<0.05) | 299 | 4.0 | 13.4 | 1.0 |
| B | ***Fazenda Ocidente*** | 1938-2017 | 78 | 894 | 12.8 | 1.00  (<0.05) | 894 | 12.8 | 12.8 | 3.2 |
| C | ***Fazenda Cachoeira D’Antas*** | 1981-2016 | 36 | 395 | 161 | 0.90  (<0.05) | 370 | 160 | 13.2 | 40.1 |
| D | ***Cachoeira dos Óculos*** | 1974-2016 | 43 | 473 | 224 | 0.89  (<0.05) | 444 | 223 | 13.0 | 55.6 |
| E | ***Governador Valadares*** | 1969-2016 | 48 | 535 | 552 | 0.91  (<0.05) | 502 | 554 | 12.5 | 138.4 |
| F | ***Resplendor*** | 1984-2004 | 21 | 242 | 645 | 0.91  (<0.05) | 240 | 643 | 12.8 | 160.8 |
| G | ***Linhares*** | 1967-1994 | 28 | 300 | 976 | 0.87  (<0.05) | 298 | 975 | 12.2 | 243.8 |
